# The Hydrostatic Pressure Distribution in the Periodontal Ligament and the Risk of Root Resorption—A Finite Element Method (FEM) Study on the Nonlinear Innovative Model

**DOI:** 10.3390/ma17071661

**Published:** 2024-04-04

**Authors:** Anna Ewa Kuc, Kamil Sybilski, Jacek Kotuła, Grzegorz Piątkowski, Beata Kowala, Joanna Lis, Szymon Saternus, Michał Sarul

**Affiliations:** 1 Department of Dentofacial Orthopedics and Orthodontics, Wroclaw Medical University, 50-425 Wroclaw, Poland; j_kotula@poczta.onet.pl (J.K.); beata.kawala@umw.edu.pl (B.K.); joanna.lis@umed.wroc.pl (J.L.); 2 Faculty of Mechanical Engineering, Military University of Technology, 00-908 Warsaw, Polandszymon.saternus@wat.edu.pl (S.S.); 3 Greg Dent Orthodontics Sp. Z O.O., 91-163 Lodz, Poland; grzegorz.piatkowski@greg-dent.pl; 4 Department of Integrated Dentistry, Wroclaw Medical University, 50-425 Wroclaw, Poland; michal.sarul@umw.edu.pl

**Keywords:** PDL hydrostatic pressure, root resorption, orthodontic treatment, FEM, incisor retraction, en masse retraction, PDL stress distribution

## Abstract

Excessive orthodontic force can induce inflammatory tooth root resorption due to sustained high stresses within the periodontal ligament (PDL). This study aimed to analyze the PDL pressures during upper incisor retraction using the en masse method with TISAD. The finite element method (FEM) ensured consistent conditions across cases. The models included bone geometry, adjacent teeth, PDL, and orthodontic hardware, analyzed with LS-Dyna. The pressure ranged from 0.37 to 2.5 kPa across the dental arch, with the central incisors bearing 55% of the load. The pressure distribution remained consistent regardless of the force or hook height. The critical pressure (4.7 kPa) was exceeded at 600–650 g force, with notable pressure (3.88 kPa) on the palatal root wall of the right central incisor. Utilizing 0.017 × 0.025 SS archwires in MBT 0.018 brackets provided good torque control and reduced the root resorption risk when forces of 180–200 g per side were applied, maintaining light to moderate stress. Triple forces may initiate resorption, highlighting the importance of nonlinear finite element analysis (FEA) for accurate oral cavity simulations.

## 1. Introduction

Orthodontic treatment employing sliding biomechanics represents the prevailing method for restoring proper occlusion. In instances of crowding, Class II malocclusion, incisor protrusion, or when preparing for surgical correction of Class III defects, extraction of the upper first premolars is frequently indicated to facilitate incisor retraction. Alternatively, in less severe cases, distalization of the entire dental arch may be warranted to restore proper occlusion and enhance the patient’s facial profile [1]. In clinical practice, orthodontists have a choice between two types of bracket slots: 0.018 and 0.022 inches. The selection of an appropriate steel archwire size depends on the chosen slot, through which the desired orthodontic movements are executed. Factors such as the archwire dimensions, hook height, and torque play a crucial role in controlling incisor inclination during arch retraction or distalization. The archwire’s stiffness aids in preventing unwanted inclinations and the occurrence of a “roller coaster” effect, which can exacerbate vertical overjet. Furthermore, in cases of significant severity, orthodontic mini-implants serve as valuable skeletal anchors, offering superior torque and anchorage control [2,3].

The integration of brackets, archwires, force vectors, and orthodontic techniques, in conjunction with the anatomy of the maxillary alveolar process, dictates orthodontic movement and potential side effects such as excessive incisor tipping, contact with the lamina dura, or root resorption, particularly in the upper incisors. The primary objective of orthodontic treatment is to attain optimal tooth displacement while minimizing adverse effects and enhancing the patient’s facial profile.

Orthodontic movement is facilitated by the inherent ability of the alveolar processes in the maxilla and mandible to undergo remodeling in response to orthodontic forces. Adhering to the principles of homeostasis governing bone apposition mediated by osteoblasts and bone resorption regulated by osteoclasts is vital for facilitating optimal bone remodeling, resulting in the formation of healthy bone tissue. The cyclical processes of resorption and apposition inherent in orthodontic interventions should be carefully managed in accordance with biomechanical principles to promote the development of healthy, well-structured bone throughout the entire therapeutic process. However, in pathological conditions, such as pathological osteolysis, inflammation plays a pivotal role in initiating and perpetuating bone destruction. Excessive activation of inflammasome–supramolecular protein complexes responsible for maturing and secreting pro-inflammatory cytokines can lead to chronic inflammation, infection spread, and uncontrolled alveolar bone loss, potentially occurring during orthodontic treatment [4].

The activation of inflammasomes may contribute to alveolar bone loss in response to orthodontic force application. This process closely correlates with the induction of periodontal inflammation. Moreover, increased orthodontic force can enhance the pro-osteoclastogenesis capacity of osteoblasts while simultaneously diminishing osteoblast activity, thereby reducing the bone formation ability, differentiation, and proliferation, and promoting osteoblast pyroptosis. Consequently, this dysregulation results in unchecked bone resorption and compromised new bone formation [5].

The application of orthodontic force generates stresses within the periodontal ligament (PDL), which, when surpassing the blood pressure in the capillary arterioles, induce hyalinization, ischemia, and necrosis of adjacent tissues, root cement, and alveolar bone. Cells near the necrotic area may initiate root resorption [6]. Hence, a correlation has been established between excessive orthodontic force, resulting in sustained high stress levels in the PDL, compromised blood flow, and orthodontically induced inflammatory tooth root resorption (OIIRR) [7].

However, the precise origins of orthodontically induced inflammatory tooth root resorption (OIIRR) remain incompletely elucidated. Its etiology is notably multifaceted and not entirely comprehended. Apart from the aforementioned excessive orthodontic forces, the various contributing factors include genetic predisposition, duration of orthodontic treatment, extent of tooth displacement, and the nature of the force applied, whether continuous or intermittent [8,9,10,11,12,13]. Additionally, root resorption may occur due to contact with the lamina dura related to the alveolar process or the incisive canal [9,14,15,16]. Notably, Kaley and Philips demonstrated a twentyfold increase in the risk of root resorption of the upper incisors due to cortical plate contact [9]. Hence, meticulous torque control and personalized planning of incisor positioning within the existing bone envelope are imperative for preserving healthy roots.

Measuring the pressure values within the periodontal ligament (PDL) resulting from orthodontic treatment and their distribution in the roots is impractical under clinical conditions. However, such values can be estimated through the finite element method (FEM) model analysis pioneered by Yettram et al. in orthodontics [17]. This method enables the simulation of complex mechanical stress scenarios within the jaw, alveolar ridge, and teeth. By analyzing the results, it becomes possible to identify the loads and locations where pressures may exceed those in the periodontal blood vessels, potentially leading to complications such as root resorption.

Finite element analysis (FEA) stands out as an exemplary approach for scrutinizing data within mathematical models. It furnishes precise, accurate, and quantifiable insights, enabling thorough evaluation and analysis across various strata. Consequently, FEA emerges as the quintessential analytical tool for assessing the stress and strain in implantology’s planned technical systems. A key characteristic of the finite element method (FEM) is its high fidelity in replicating physical properties between the real-world structure and the FEM model. However, it warrants acknowledgment that oversimplification of the geometry of the object under scrutiny poses a potential risk of yielding inconsistent evaluation outcomes [18].

### Objective

The objective of this study was to analyze the pressure exerted on the periodontium of tooth roots during the retraction of upper incisors using the en masse method with Temporary Anchorage Devices (TADs) following the extraction of first premolars using a high-standard innovative finite element model. Additionally, this study aimed to assess the pressure during the retraction of the entire dental arch, considering the applied force and its vector on the 0.017 × 0.025 stainless steel (SS) archwire in 0.018 MBT prescription slot brackets, utilizing finite element model analysis.

## 2. Materials and Methods

To achieve the objectives, a research methodology centered on numerical analyses, specifically employing the finite element method, was chosen. This approach facilitated the precise replication of conditions across all cases under examination. Ensuring the uniformity of conditions is essential for meaningful comparative analysis of selected parameters.

### 2.1. Construction of a Numerical Model

One of the primary objectives of this study was to faithfully replicate real patient conditions while ensuring the proper consideration of cranial stiffness in the preservation of dental and periodontal structures. To achieve this, a numerical model was constructed based on computed tomography (CT) scans of the cranial region and intraoral scanning. DICOM files derived from the CT scans were processed into STL files utilizing the MIMICS system (Materialise, Leuven, Belgium) [19]. During this process, emphasis was placed on delineating three distinct geometrical groups: compact bone, spongy bone, and teeth (inclusive of their roots). To enhance the geometric precision, each layer of the DICOM file was meticulously outlined based on grayscale values corresponding to individual structures.

**Figure 1:**
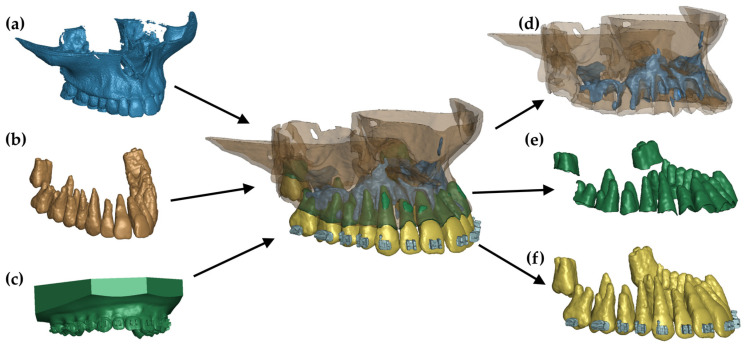
FE model: (**a**) geometry extracted from a CT image, (**b**) teeth extracted from a CT image, (**c**) scan of a dental arch with brackets, (**d**) combination of cortical and cancellous bone, (**e**) finite elements of the periodontium, and (**f**) teeth with brackets.

The result of this process was a surface mesh consisting of triangles representing the outer contour of the cortical bone (Figure 1a) and teeth (Figure 1b). The cancellous bone was delineated by identifying the enclosed volumes within the cortical bone contour.

In addition, data from intraoral scans of dental arches, including those with fixed brackets, were included in the construction of the numerical model. The dental arch scan (Figure 1c) was aligned with the teeth delineated from the CT scans, and then STL models of the brackets provided by the manufacturer were imported and individually positioned for each tooth.

In the subsequent phase, the contours delineating the cortical bone, cancellous bone, teeth, and brackets (see Figure 1d) were populated with a mesh of 3D elements using the Hypermesh system (Altair, Troy, MI, USA). Tetragonal elements were selected for this purpose. Taking advantage of the tooth and bone geometries, the periodontal ligament (PDL) was modeled using hexahedral elements, with an assumed consistent thickness of 0.25 mm [20]. The average mesh size was set to 0.32 mm.

**Figure 2:**
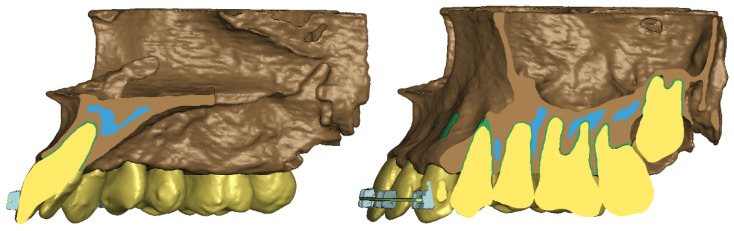
Cross-section: numerical model.

**Figure 3:**
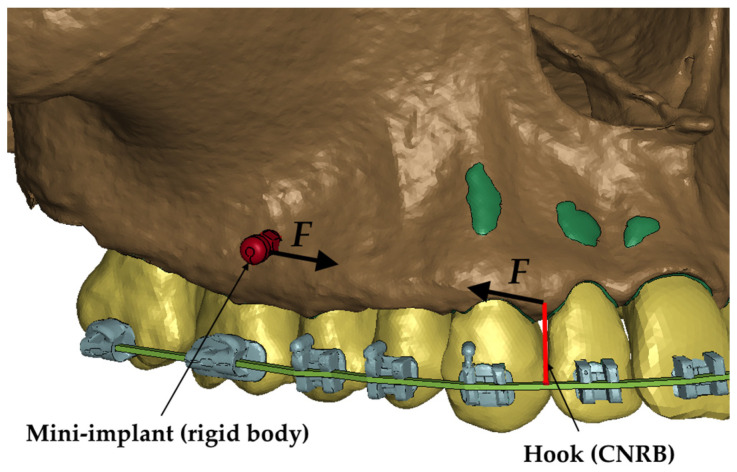
Load representation in the numerical model.

**Figure 4:**
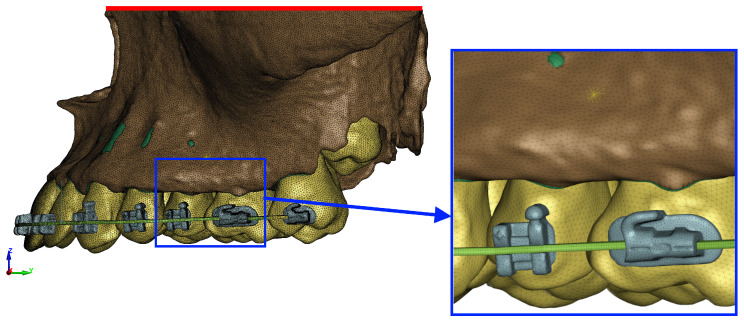
Boundary condition in the numerical model (red line represents constrained surface) and finite elements (average mesh size = 0.32 mm).

The final stage of replicating the patient’s anatomy was to model the mini-implant and wire. The wire was shaped based on the bracket geometry, delineating a rectangular cross-section along a curve connecting successive bracket openings. Hexahedral elements were used to reproduce this geometry in the numerical model. Consequently, a comprehensive numerical model comprising 3D elements was generated, as depicted in Figure 2, Figure 3 and Figure 4.

### 2.2. Material Modeling

In the numerical model, based on the preliminary simulations, it was verified that the wire, brackets, teeth, and bones would be subjected to low loads and that there would be no significant stresses in these components. It was therefore assumed that these structures would be modeled using an isotropic, linear elastic constitutive model. The brackets and wire were made of steel. The material data for the above components are given in Table 1.

The most heavily loaded component, and at the same time, the component of greatest interest to the authors, was the periodontium (PDL). Therefore, the hyperelastic Ogden model was used to model the PDL. This model assumes that the behaviour of the material can be described by the strain energy function, from which the stress–strain relationship can be derived. In the Ogden model, the strain energy function is defined by the function:

(1) $$W=\sum_{i=1}^{3}\sum_{j=1}^{n}\frac{\mu_{j}}{\alpha_{j}}\left(\lambda_{i}^{\alpha }j-1\right)+K\left(J-1-\ln J\right)$$

where *W* is the strain energy potential, *λ_i_* is the main deviant stretches, *µ_i_* and *α_i_* are material parameters, *J* is the determinant of the elastic strain gradient, and *K* is the volume modulus. The bulk modulus is calculated using the values of Poisson’s ratio and Young’s Modulus. The parameters presented in Table 2 [23] were used to describe the behavior of the PDL.

### 2.3. Restraint and Load Conditions, Contacts

In the developed numerical model, the mini-implant’s stiffness significantly sur-passed that of the surrounding structure. Consequently, the mini-implant (see Figure 3) was represented as a rigid element constrained to the adjacent nodes. Similarly, a hook attached to the wire was modeled using the same approach. The rigid element, called a Constrained Nodal Rigid Body (CNRB), encompasses all six degrees of freedom. In practical scenarios, an elastic element typically bridges the gap between the mini-implant and the hook, generating tension of a predetermined magnitude. Thus, within the numerical model, a coordinate system was established with the x-axis aligned between the CNRB nodes, corresponding to the actual positions of the fixtures. Along this defined x-axis, a force (F) was applied, as illustrated in Figure 3. The force was therefore applied between the upper node of the hook (Figure 3) and the outer node of the mini-implant. This procedure was replicated on both sides of the numerical model.

The numerical model was fixed over the entire surface of the upper part (Figure 4—red color). All the translational degrees of freedom of the nodes lying on this surface have been fixed.

Appropriate contacts were defined between the touching components. A penalty function-based contact with a friction coefficient of 0.6 was defined between the wire and the brackets [24]. The tooth brackets were attached using a tied contact available in the LS-Dyna system. The same contact was defined between the teeth and periodontium, periodontium and bone. All the analyses were static and were performed using an implicit integration step in the LS-Dyna system. The individual loads changed linearly during the analysis from 0 at t = 0 to the maximum value at t = 1.

## 3. Results

The hydrostatic pressure values σ_h_, along with the distribution map within the periodontal ligament (PDL) during en masse retraction using Temporary Skeletal Anchorage Devices (TISADs) subsequent to the extraction of first premolars in the 0.018 slot on the 0.017 × 0.025 stainless steel (SS) arch, employing various hook heights and force magnitudes, are presented in Table 3, Table 4, Table 5 and Table 6. Table 3 depicts the comprehensive distribution and pressure values σ_h_ across the entire dental arch. Furthermore, Table 4 delineates the pressure σ_h_ distribution and values within the PDL of the roots of the upper central incisors, while Table 5 elucidates the corresponding data for the upper lateral incisors. Additionally, Table 6 portrays the distribution and pressure σ_h_ values within the PDL of the canine roots.

**Figure 5:**
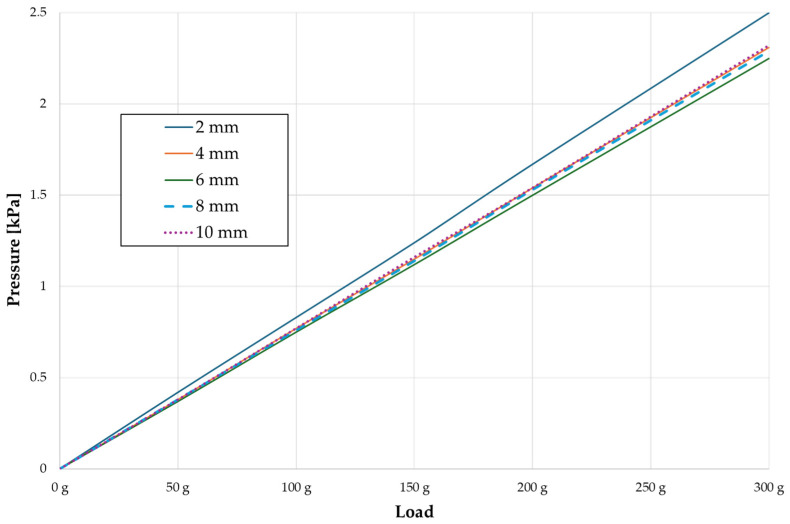
Pressure σ_h_ versus load applied in the PDL for the entire dental arch.

Across a force spectrum ranging from 50 g to 300 g, the pressure σ_h_ values for the entire dental arch vary between 0.37 kPa and 2.5 kPa (see Table 3). Notably, the pressure σ_h_ values demonstrate a linear correlation with the increment of applied force (refer to Figure 5). However, marginal differences are observed in the pressure values σ_h_ corresponding to different hook heights for a given force magnitude, which are clinically insignificant. Notably, in all the described scenarios, the lowest pressure σ_h_ is observed for a 6 mm hook height, while the highest is noted for a 2 mm hook height.

The pressure σ_h_ exerted within the periodontium of the central incisors (refer to Table 4) spans from 0.23 kPa to 1.54 kPa, exhibiting a linear relationship with the applied force. In contrast to the entire dental arch, the minimum pressure σ_h_ values are observed at the lowest hook height of 2 mm, progressively escalating with an increased hook height. Across all instances, the pressure σ_h_ values exerted on the central incisors represent approximately 55% of the total pressure values observed within the periodontium of the entire dental arch.

The pressure σ_h_ in the periodontium of the lateral incisors (Table 5) is approximately 45% of the pressure of the full arch and ranges from 0.18 kPa to 1.14 kPa, with a linear distribution. Unlike the entire arch and central incisors, the lowest values are recorded at the highest hook height of 10 mm and increase as the hook height decreases.

In the case of the canines (Table 6), the pressure values σ_h_ are the highest and constitute approximately 75% of the value of the entire pressure in the PDL of a full arch. The values range from 0.28 to 1.83 kPa. The relationships are equivalent for the lateral incisors.

During the comparative analysis of the hydrostatic pressure σ_h_ within the periodontal ligament (PDL) across both the entire dental arch and individual teeth, the distribution map remains consistent irrespective of the applied force or hook height. Therefore, the distribution map remains constant regardless of the force vector utilized, with only the proportional values varying (refer to Table 3, Table 4, Table 5 and Table 6).

For the central incisors, the highest pressure σ_h_ values are observed along the palatal aspect, with consistent accumulation predominantly in the lower half across all cases. Additionally, the apical third of the roots typically resides within the neutral pressure zone, except for minor points at the apices where higher pressures concentrate. Notably, higher pressure σ_h_ values are generally observed for the right incisor compared to the left incisor (see Table 5).

Regarding the lateral incisors, pressure σ_h_ accumulation predominantly occurs in the cervical region along the distal palatal wall and at specific points near the apices, while the remaining portions of the roots reside within the neutral pressure zone (refer to Table 5).

However, the majority of the canines are located in the neutral zone, with a single place of pressure σ_h_ accumulation in the cervical area of the palatal wall. The left canine has slightly higher values (Table 6).

**Figure 6:**
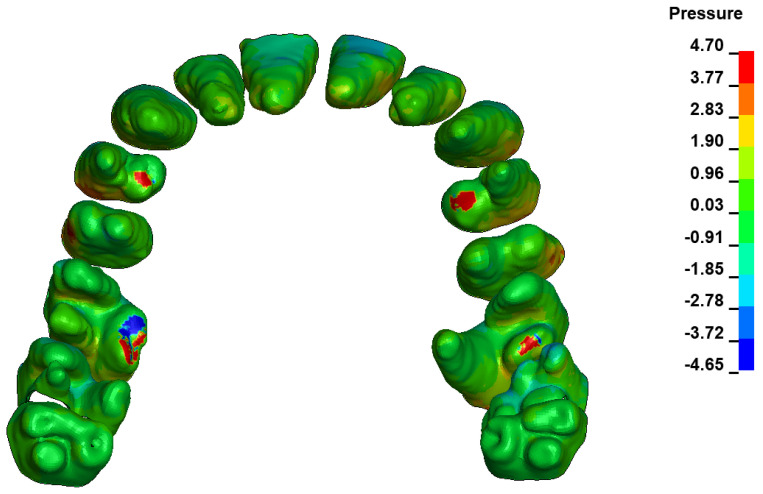
Pressure σ_h_ [kPa] in the PDL for load = 642 g—full arch.

**Figure 7:**
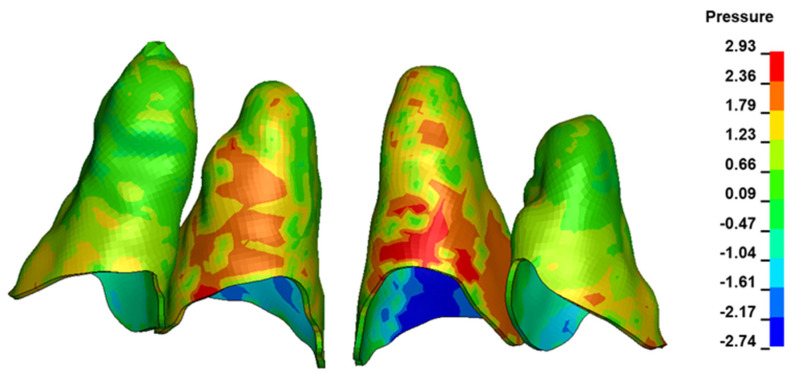
Pressure σ_h_ [kPa] in the PDL for load = 642 g—central incisors.

The critical value of 4.7 kPa is exceeded for the full dental arch with a force of 642 g and is concentrated on the upper roots of the first molars, reaching at the same time in the anterior segment 2.93 kPa, which accumulates mainly on the right central incisor in the area of the lower palatal half root walls (Figure 6 and Figure 7).

The pressure σ_h_ values, along with the distribution map generated within the periodontal ligament (PDL) during distalization of the entire dental arch using Temporary Skeletal Anchorage Devices (TISADs) in the 0.018 slot on the 0.017 × 0.025 stainless steel (SS) arch employing various hook heights and force magnitudes, are delineated in Table 7, Table 8, Table 9 and Table 10. Table 7 presents the comprehensive distribution and pressure σ_h_ values across the entire dental arch. Furthermore, Table 8 depicts the pressure distribution and values within the PDL for the roots of the upper central incisors, while Table 9 elucidates the corresponding data for the upper lateral incisors. Additionally, Table 10 portrays the distribution and pressure σ_h_ values within the PDL for the roots of the canines and first premolars.

**Figure 8:**
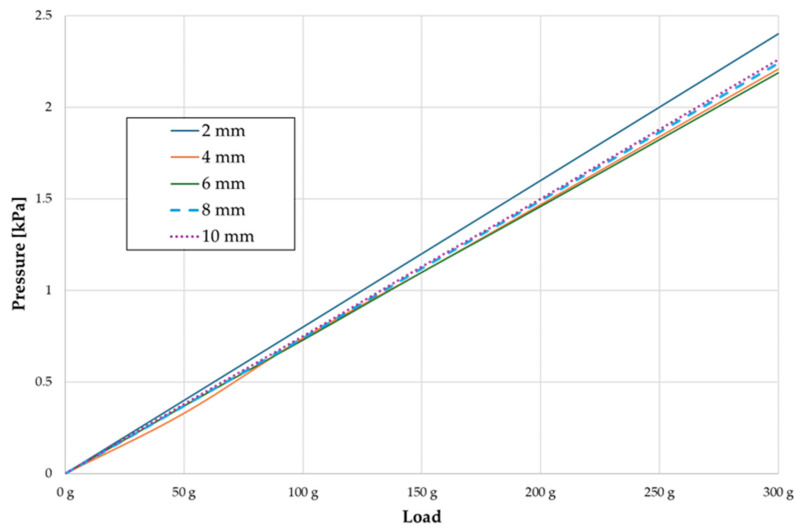
Pressure σ_h_ [kPa] versus load applied in the PDL for the entire dental arch.

In the range of applied forces from 50 g to 300 g, the pressure σ_h_ values for the entire dental arch are very similar and range from 0.33 kPa to 2.4 kPa (Table 7). The pressure σ_h_ values also increase linearly with the increase in the applied force (Figure 8). However, the differences at the level of one force value at different hook heights are even more minimal. All the dependencies are as above. The pressure σ_h_ values are approximately 9–10% lower everywhere. However, the pressure σ_h_ distribution map is the same in all cases.

## 4. Discussion

Side effects are common occurrences during orthodontic treatment, with root resorption (OIRR) being a frequent concern. The initial signs of resorption typically become evident under a microscope approximately two weeks after treatment initiation [25], while radiological manifestations may appear several months into treatment [26,27]. Although previous studies suggest that the bracket size and archwire selection do not significantly influence the occurrence of resorption [28,29], contact with the cortical lamina is a well-documented risk factor for severe root resorption [9].

The most desirable outcome of orthodontic treatment is achieving axial movement of the teeth while maintaining appropriate torque control to minimize the risk of root tip movement in the opposite direction. Often, achieving this goal necessitates additional interventions such as bilateral corticotomy, intrusion archwires or skeletal anchorage. This is particularly important due to the approximately 9–10% difference in torque between the working archwires, such as 0.0190.025 stainless steel wires in 0.022 brackets, and the commonly used archwires, such as 0.0160.022 wires in brackets with a 0.018 slot [30].

The finite element model (FEM) represents a modern and invaluable tool for assessing the risk of root resorption associated with necrosis induced by capillary lumen closure due to stress and increased pressure σ_h_ within the periodontal ligament (PDL) resulting from orthodontic force application. The reliability of this method varies based on the quality of the model preparation. One of the salient characteristics of FEM is the near physical similarity among the real structure as well as its FEM. However, unnecessary simplification of geometry will invariably lead to inconsistent results [18]. Linear models, which have been the focus of much research [31,32,33,34,35,36,37,38,39,40], lack specificity and fail to accurately represent bodily tissues compared to nonlinear models [8]. In our proposed research, we utilized a high-quality, innovative, fully flexible nonlinear model based on Cone Beam Computed Tomography (CBCT) scans of a typical patient with Class II/1 mal-occlusion requiring extensive retraction. This model includes comprehensive anatomy, encompassing the vestibular and palatal cortical plates, alveolar laminae, interdental septa, and the incisive canal lamina, with all the structures interacting as they would in vivo. By ensuring all the stresses are accurately transmitted to the surrounding structures, we can confidently assume the high reliability of our measurements. The presence of fenestrations in the vestibular plate of the maxillary alveolar process, subsequent to tooth movement for alignment and insertion of a 0.017 × 0.025 stainless steel (SS) working wire into orthodontic brackets, serves as a clear indication of the consequences of inappropriate orthodontic treatment methods. Failure to respect the bone envelope and reduce tooth material while expanding the dental arch can lead to such complications.

The periodontal ligament (PDL) plays a crucial role in orthodontic movement [41]. Therefore, the model should accurately represent the movement of teeth through the periodontium and various bone layers with appropriate elastic moduli, ensuring interconnectedness. In our analysis, we focused on hydrostatic stress σ_h_ rather than von Mises stress σ_vM_ and minimum principal stress σ_3_, as the former is directly associated with the formation of resorption lacunae in tooth roots [8,37]. Research has demonstrated that locations where the hydrostatic pressure σ_h_ exceeds 4.7 kPa correspond to areas of root resorption observed via electron microscopy. Conversely, areas exhibiting expansion during model simulation did not exhibit active resorption [31]. Clinical studies have not revealed resorption defects in locations with high von Mises σ_vM_ values or minimum principal stress σ_3_ as determined by finite element analysis [8].

This study focused on analysing 0.018-inch slot brackets. It demonstrated a linear relationship between the applied force and the resulting pressure σ_h_ in the periodontal ligament (PDL) of tooth roots, with increasing force leading to higher hydrostatic pressure σ_h_. These findings align with previous studies by Owman-Moll [42] and Maltha [43] but contradict those of Cas [44] and Chan and Darendeliler [45]. This study also explored the percentage distribution of pressure among different tooth groups: 55% on the central incisors, 45% on the lateral incisors, and 75% on the canines. Notably, this distribution has not been reported in the prior literature. Furthermore, no significant differences were observed in the hydrostatic pressure σ_h_ values or their distribution maps concerning the height of the hook, whether during en masse retraction or the distalization of the entire arch. These findings are particularly intriguing, as one might expect significant changes in the movement mechanics when extracting teeth. However, our study revealed that during en masse retraction with appropriately low forces, the distribution of stresses σ_h_ around the roots of the anterior teeth remains largely unchanged. It is worth noting that due to our utilization of newer modeling techniques, comparisons with studies using linear models, measuring von Mises stresses σ_vM_ or minimum principal stress σ_3_ [31,34,35,46,47,48], or examining PDL deformation [48] are challenging.

The absence of significant high values at the incisor apices and the relatively even distribution of the roots along the palatal wall suggest effective torque control and axial displacement of the teeth, irrespective of the applied force vector. These conclusions differ from those of Tominaga et al., whose study revealed discrepancies in the axial displacement among the front teeth segment at a hook height of 5 mm positioned behind the lateral incisor, as well as tilting at hook heights of 0 mm and 10 mm [49,50]. Such variations may stem from the utilization of an individualized model that accurately reflects the anatomical conditions under which specific biomechanics are employed and all stresses are transferred to adjacent structures. Additionally, in the presented model, the applied force may result in the bending of both the root and the interdental septum plate, which may result in a different stress σ_h_ distribution than in simplified models. Further research is warranted to confirm or refute this hypothesis. 

Based on the study findings, it can be inferred that a threefold increase in the optimal orthodontic force for en masse retraction, ideally ranging from 180 to 200 g per side, leads to areas where the pressure σ_h_ within the periodontal ligament (PDL) exceeds 4.7 kPa. This poses a significant risk of root resorption due to the complete occlusion of capillaries, leading to root necrosis and subsequent resorption [37]. It is conceivable that during a two-stage retraction of a segment comprising four incisors, a force less than three times the applied value may still result in capillary occlusion, as the force is distributed over a smaller number of teeth. However, further research is necessary to conclusively validate this hypothesis.

Upon analysis of the above results, it becomes evident that meticulous control of the forces and torques acting on the incisor roots during torque control is paramount in preventing tooth root tip resorption. In our model, even under axial movement, substantial forces are evenly distributed across the surface, locally remaining below the critical value σ_h_ of 4.7 kPa. Optimal orthodontic forces, ranging from 180 to 200 g per side, result in pressure σh values slightly exceeding half of the critical threshold. This level of pressure may stimulate physiological bone resorption, facilitating orthodontic movement without adverse effects by modulating blood flow in the capillaries without complete occlusion. The present study reveals that lateral incisors experience heightened pressure solely in the cervical area, remaining free from significant hydrostatic stresses σ_h_ on both the palatal and labial aspects. However, despite this, resorption of the four upper incisors is a common occurrence in clinical practice. Nevertheless, based on our findings, it can be inferred that such occurrences are less probable when utilizing a 0.017 × 0.025 archwire in a 0.018 slot with the force values specified by the authors. In this scenario, there is no abrupt increase in hydrostatic pressure σ_h_ in the apical region, which could initiate the formation of resorption defects. These results are applicable to both en masse retraction treatment following premolar extraction and during full arch distalization.

Brackets featuring a 0.018 slot were replaced in the initial phase of treatment due to their inadequate size, which is undesirable when creating ample clearance in the brackets. Additionally, their use led to increased friction during the sliding mechanics and insufficient space between the working arch and the bracket slot. Nevertheless, recent studies indicate that these brackets offer excellent torque control. Moreover, the inconvenience associated with their use in the initial alignment phase may be mitigated in the current era of high-quality, thin 0.014 A-NiTi archwires. These brackets and archwires possess significant potential to minimize the severe root resorption attributed to ischemia and excessive tipping, thereby reducing the likelihood of unintentional contact with the cortical lamina, particularly the vestibular lamina.

In their randomized studies comparing the effectiveness and treatment quality of both 0.018 and 0.022 brackets, Yassir et al. observed no significant differences in treatment outcomes [51]. Therefore, considering the hydrostatic pressure σ_h_ distribution described above, it may be reasonable to regard these brackets as viable alternatives to mitigate the risk of root resorption, particularly in individuals with thin ridges and other root resorption risk factors where precise torque control is essential. It can also be taken into account that resorption during orthodontic treatment may be caused by nickel–titanium wires or rectangular wires, which may have enhanced energy exceeding the threshold values [52]. Over the last few years, research has focused on clear aligners and no research has been conducted on the problem we are analyzing. Other previous studies concerned a similar method but on different sized arches, which makes comparison impossible. In the research conducted by Ruenpol on the en masse retraction of the upper anterior tooth segment in 0.018 slot brackets on thinner 0.016 × 0.022 SS arches, the greatest stresses were found in the central part of the roots, while the greatest expansions were found in the apical third of the maxillary lateral incisors [48]. In his research, Taminaga noticed that the axial displacement of teeth does not occur when force is applied at the level of the center of resistance. His analysis showed that in the 0.018 × 0.025 SS wire, the translational movement takes place at a level of 2.2 mm lower than the resistance center toward the incisal edge, while in the 0.016 × 0.022 arc, it is 3.8 mm higher than the resistance center toward the apex [50]. This does not agree with our results, in which the 0.018 slot on 0.017 × 0.025 SS showed good torque control regardless of the amount of applied force. In turn, Singh’s research in 0.022 slot brackets on 0.019 × 0.025 SS wire showed that the stress value in the cortical bone around the central and lateral incisors increases linearly with the increase in the height of the hook. Additionally, the authors noticed high stress values around the canines and associated that with the fact that they are surrounded mainly by cortical bone. They also showed that as the height of the hook increases, the stresses decrease at the canines and increase at the incisors [47]. Our results indicate that the new type of modeling may provide different results in terms of the application and point of application of the optimal force. Moreover, the use of our tooth retraction parameters means that the force value has a large tolerance range before it exceeds the optimum and also provides excellent torque control.

## 5. Conclusions

1. Optimally, 0.017 × 0.025 SS archwires in MBT 0.018 brackets provide excellent torque control, leading to precise axial displacement of the teeth.
2. When applying optimal forces of 180–200 g/side, there is no risk of root tip resorption due to the even distribution of light and medium hydrostatic pressure σ_h_ in the periodontal ligament (PDL).
3. The application of triple orthodontic forces (600–640 g/side) can initiate the resorption process by occluding the capillaries.
4. Attempting to level the dental arch with a significant dentoalveolar discrepancy may result in fenestrations of the vestibular plate of the alveolar process.
5. High-quality nonlinear models for finite element analysis (FES) are recommended to ensure reliable, comparable, and realistic simulations closely resembling the oral cavity conditions.

### Limitations

The presented model assumes passive insertion of the wire into the brackets after full leveling.

## Figures and Tables

**Table 1:** Material data used to describe the material behavior.

Component	Young’s Modulus [MPa]	Poisson’s Ratio
Steel	210,000	0.30
Tooth [21]	18,600	0.31
Cortical bone [22]	13,700	0.30
Cancellous [22]	2000	0.30

**Table 2:** Parameters used for describing the PDL.

*µ*_1_ [MPa]	*α*_1_ [MPa]	Poisson’s Ratio
2.5 × 10^−3^	150	0.46

**Table 3:** Pressure σ_h_ [kPa] distribution in the PDL—entire dental arch.

50 g	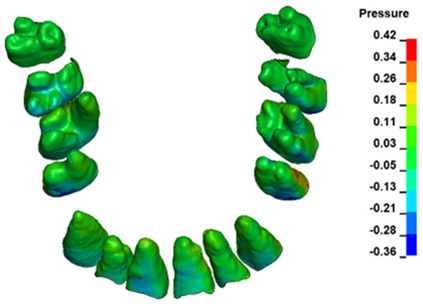	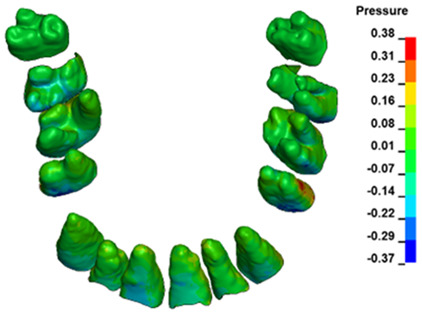
	h = 2 mm	h = 4 mm
	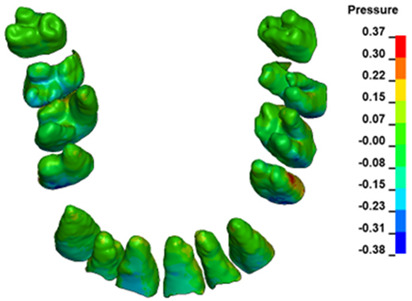	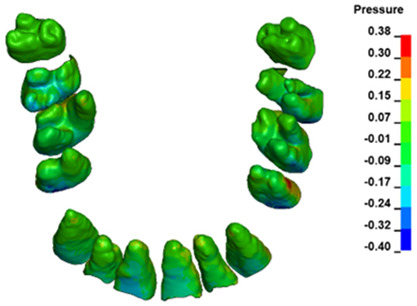
	h = 6 mm	h = 8 mm
	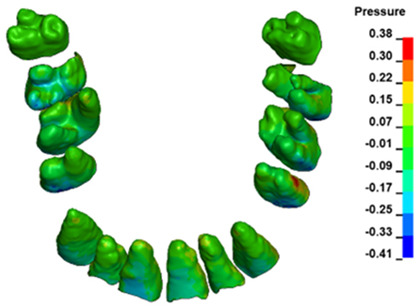	
	h = 10 mm	
100 g	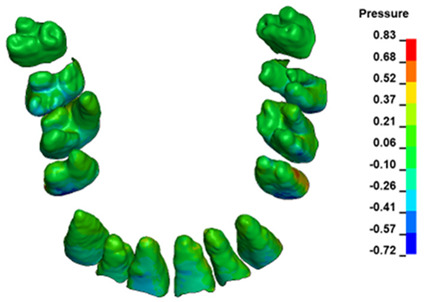	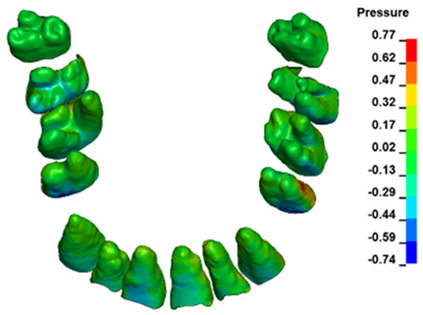
	h = 2 mm	h = 4 mm
	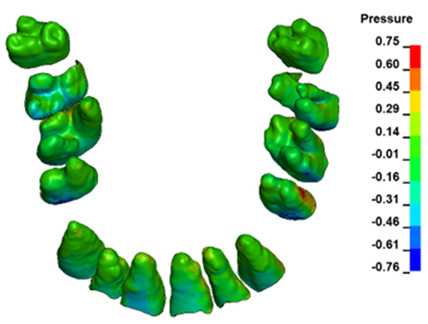	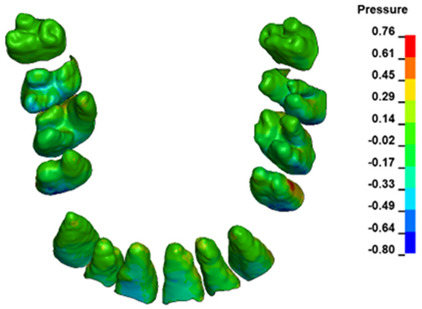
	h = 6 mm	h = 8 mm
	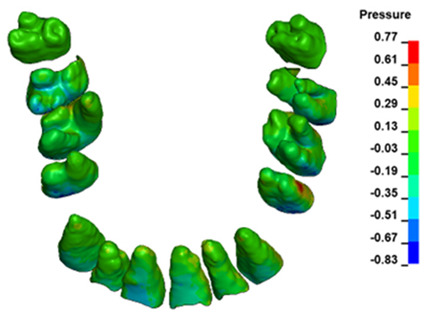	
	h = 10 mm	
150 g	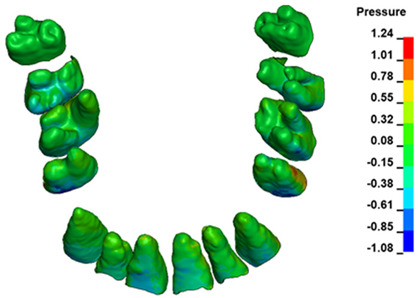	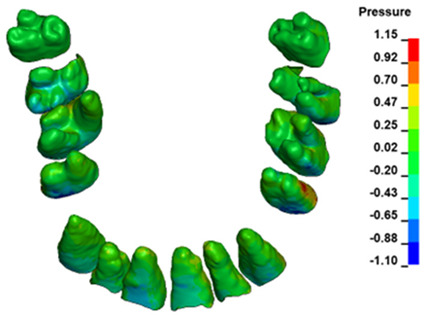
	h = 2 mm	h = 4 mm
	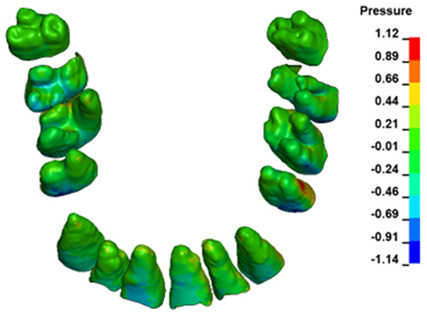	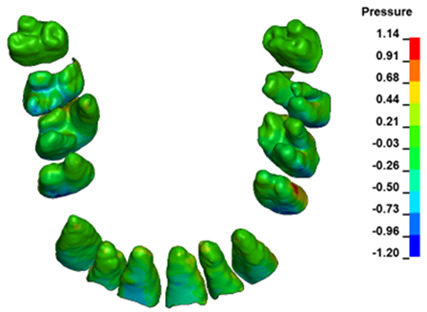
	h = 6 mm	h = 8 mm
	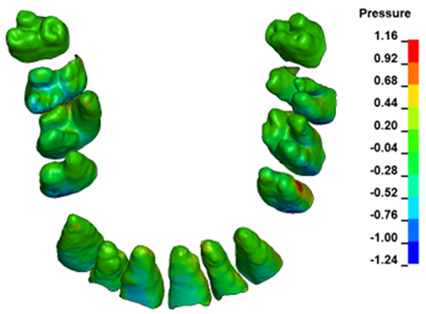	
	h = 10 mm	
200 g	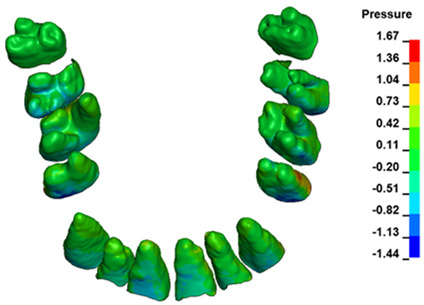	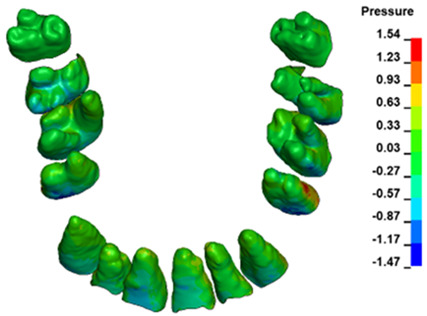
	h = 2 mm	h = 4 mm
	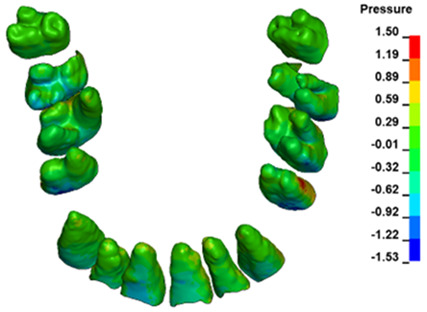	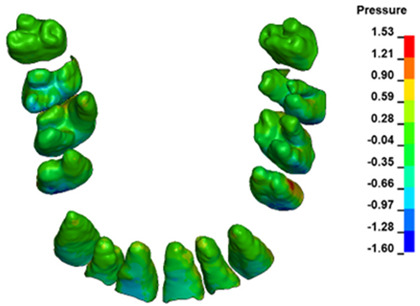
	h = 6 mm	h = 8 mm
	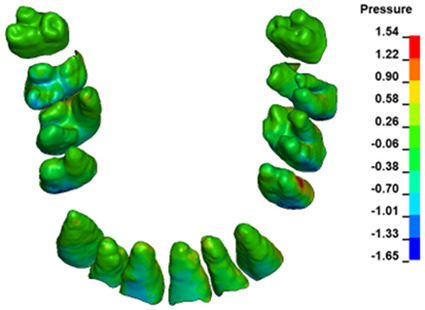	
	h = 10 mm	
300 g	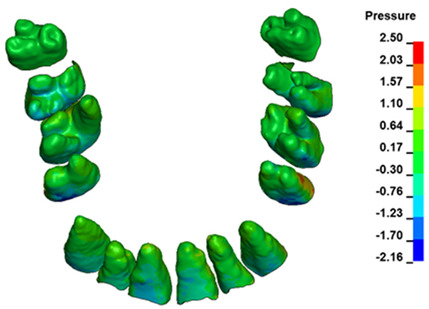	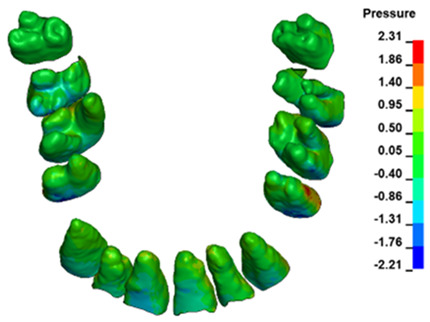
	h = 2 mm	h = 4 mm
	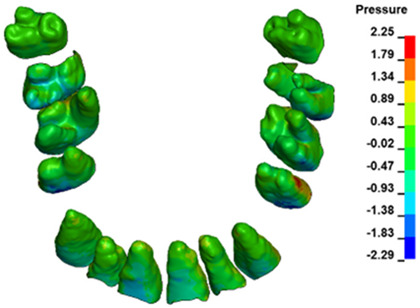	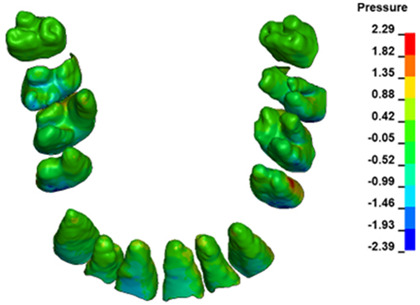
	h = 6 mm	h = 8 mm
	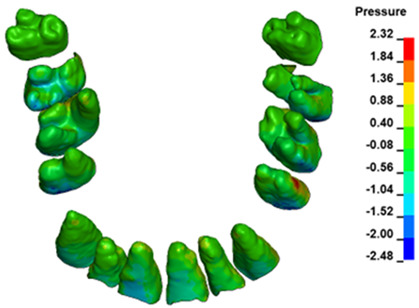	
	h = 10 mm	

**Table 4:** Pressure σ_h_ [kPa] distribution in the PDL—central incisors.

50 g	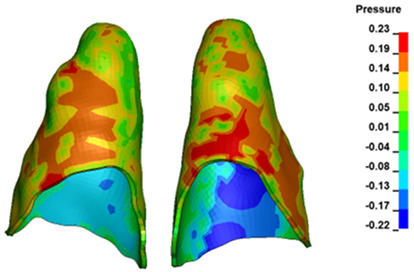	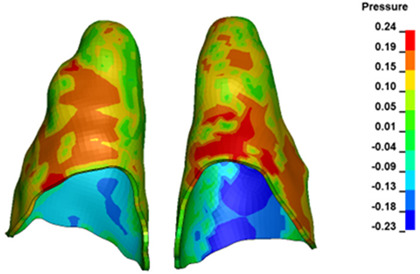
	h = 2 mm	h = 4 mm
	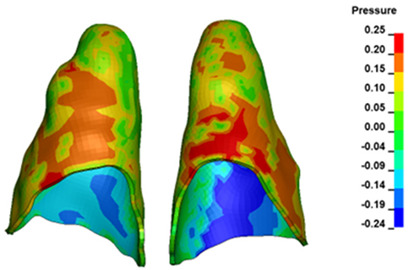	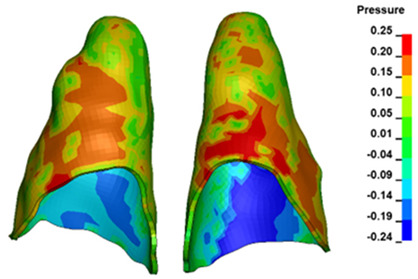
	h = 6 mm	h = 8 mm
	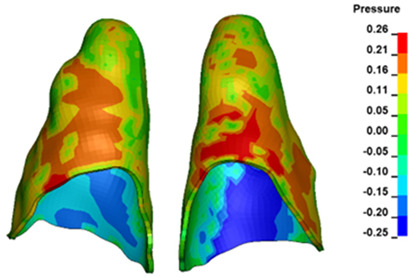	
	h = 10 mm	
100 g	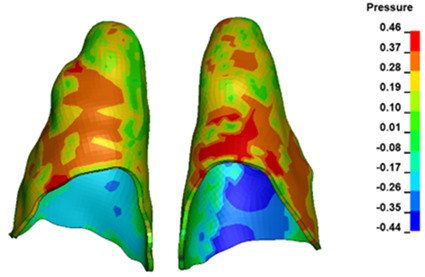	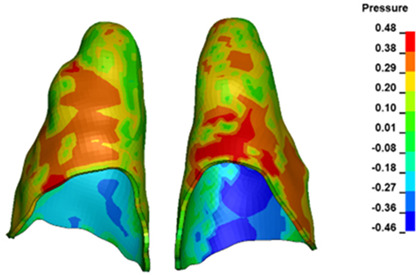
	h = 2 mm	h = 4 mm
	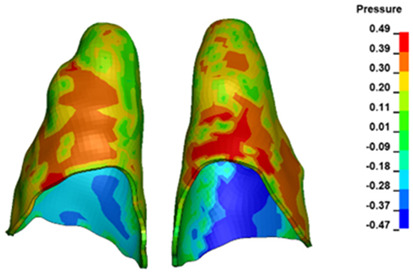	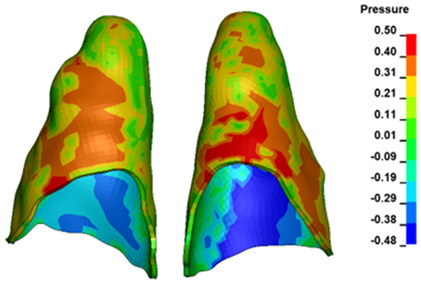
	h = 6 mm	h = 8 mm
	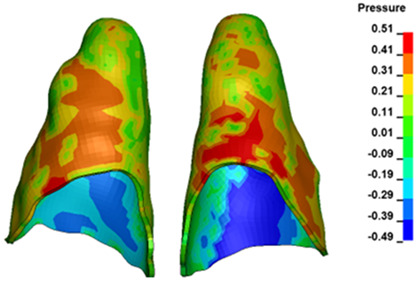	
	h = 10 mm	
150 g	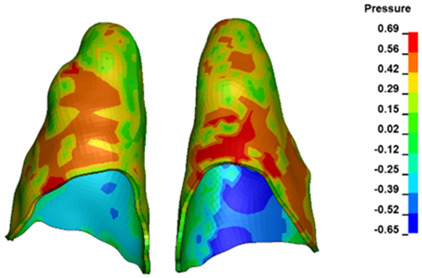	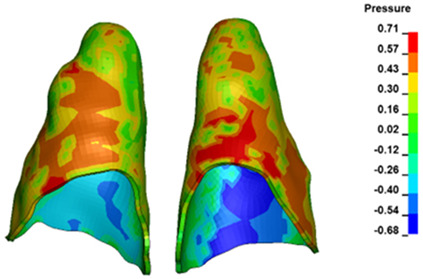
	h = 2 mm	h = 4 mm
	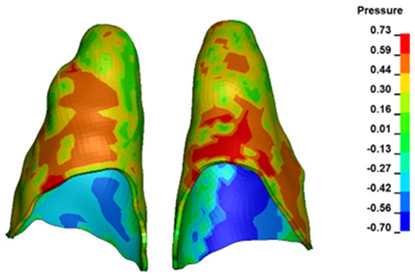	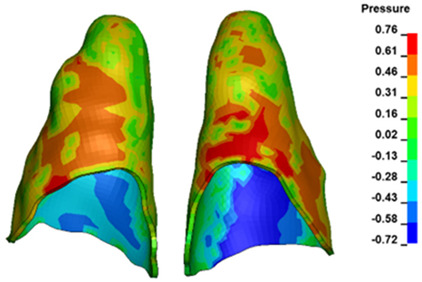
	h = 6 mm	h = 8 mm
	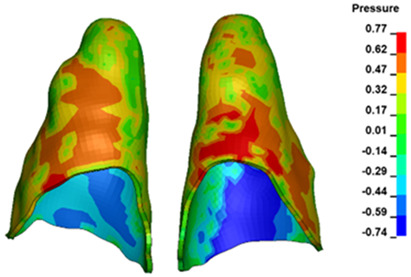	
	h = 10 mm	
200 g	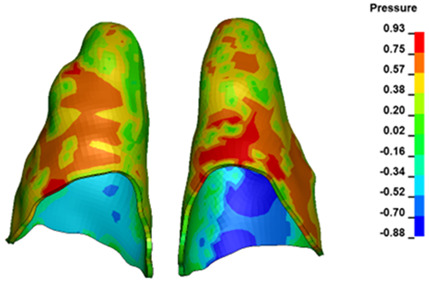	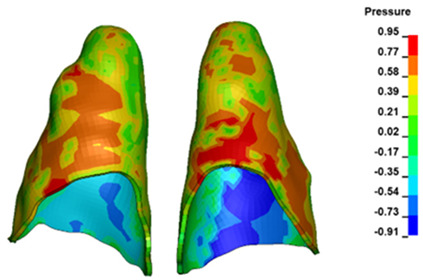
	h = 2 mm	h = 4 mm
	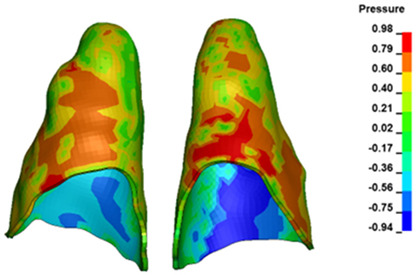	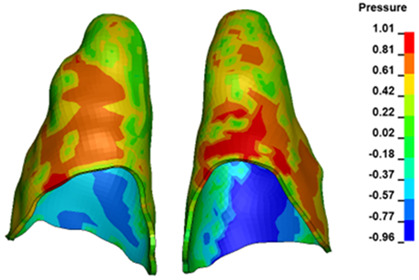
	h = 6 mm	h = 8 mm
	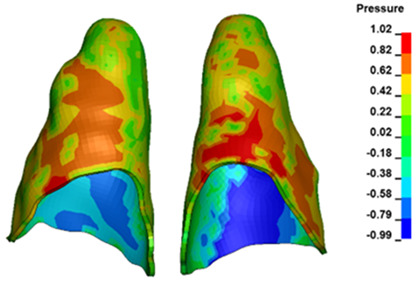	
	h = 10 mm	
300 g	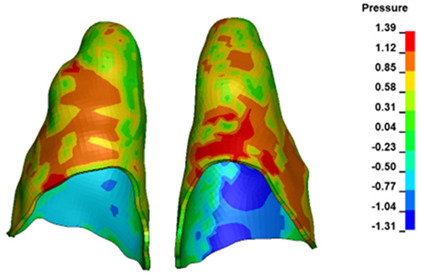	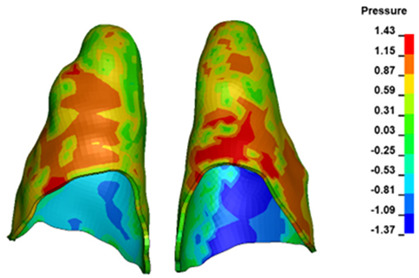
	h = 2 mm	h = 4 mm
	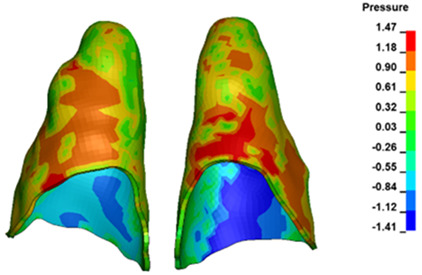	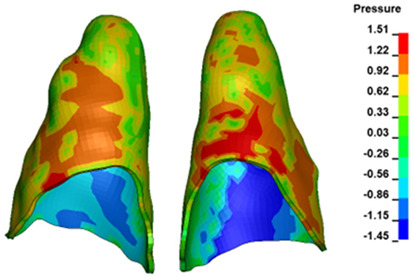
	h = 6 mm	h = 8 mm
	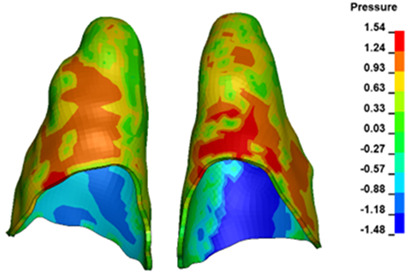	
	h = 10 mm	

**Table 5:** Pressure σ_h_ [kPa] distribution in the PDL—lateral incisors.

50 g	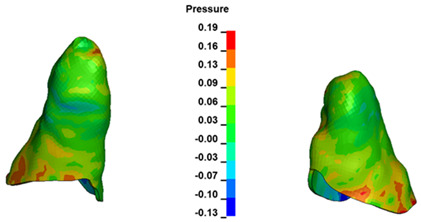	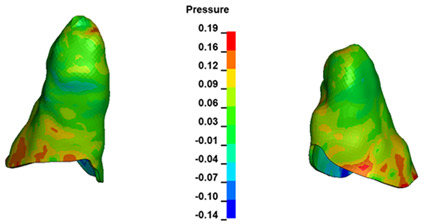
	h = 2 mm	h = 4 mm
	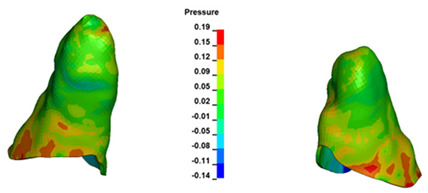	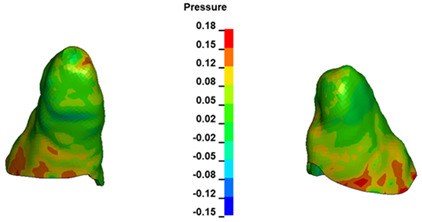
	h = 6 mm	h = 8 mm
	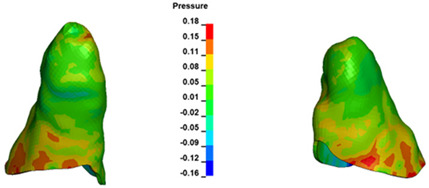	
	h = 10 mm	
100 g	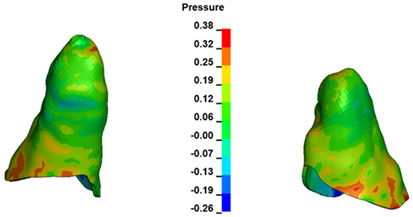	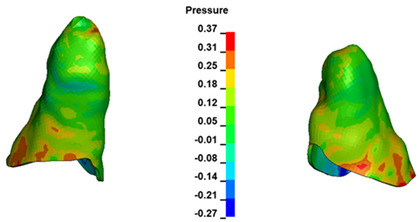
	h = 2 mm	h = 4 mm
	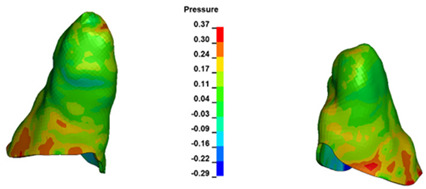	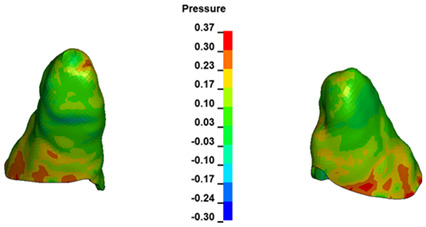
	h = 6 mm	h = 8 mm
	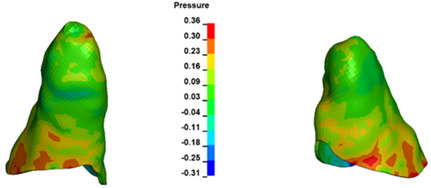	
	h = 10 mm	
150 g	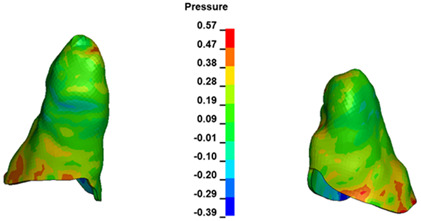	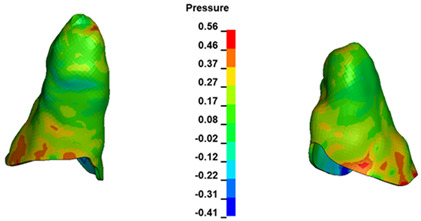
	h = 2 mm	h = 4 mm
	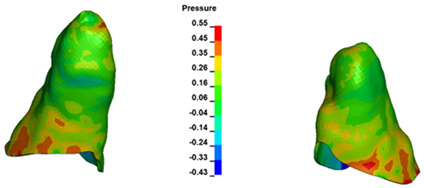	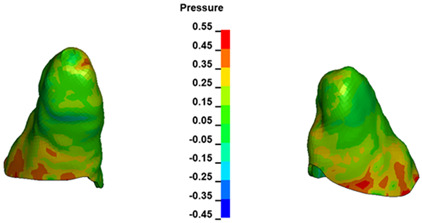
	h = 6 mm	h = 8 mm
	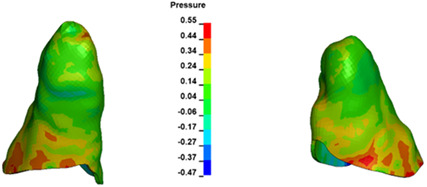	
	h = 10 mm	
200 g	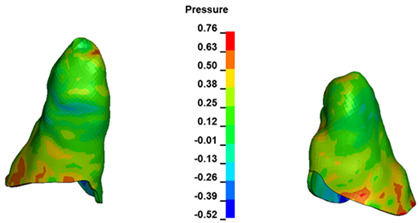	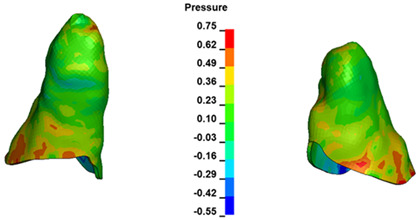
	h = 2 mm	h = 4 mm
	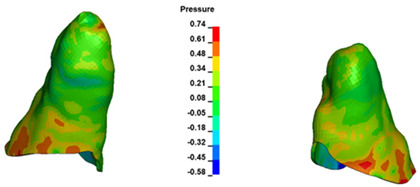	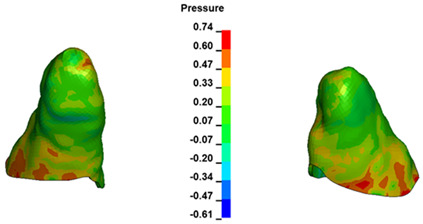
	h = 6 mm	h = 8 mm
	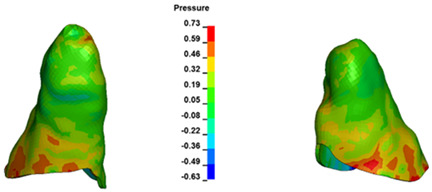	
	h = 10 mm	
300 g	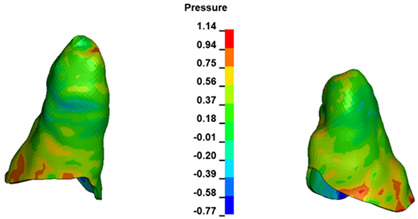	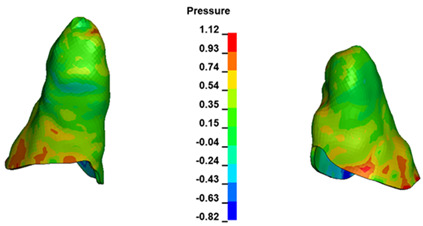
	h = 2 mm	h = 4 mm
	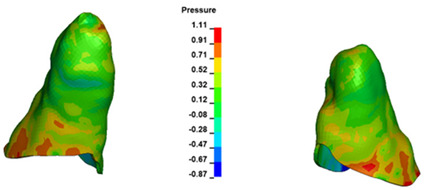	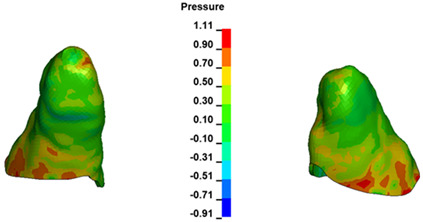
	h = 6 mm	h = 8 mm
	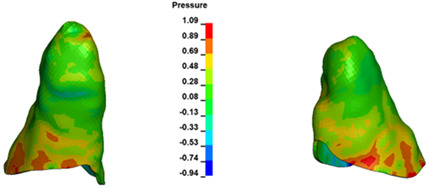	
	h = 10 mm	

**Table 6:** Pressure σ_h_ [kPa] distribution in the PDL—canines.

50 g	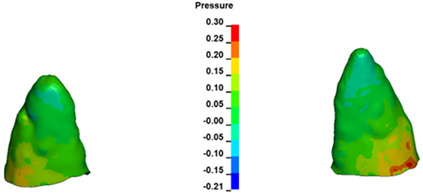	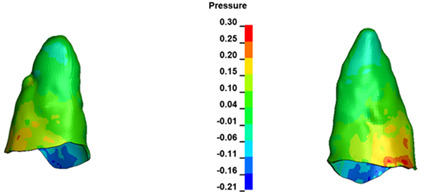
	h = 2 mm	h = 4 mm
	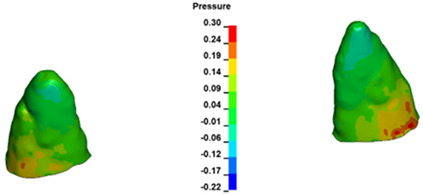	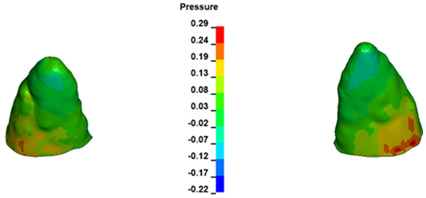
	h = 6 mm	h = 8 mm
	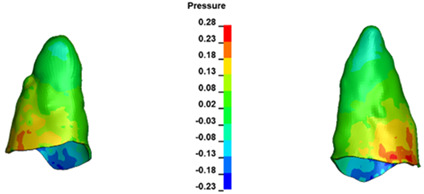	
	h = 10 mm	
100 g	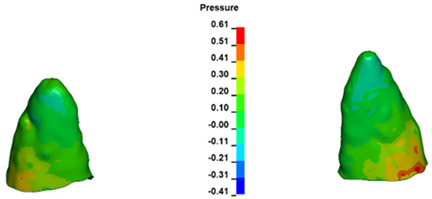	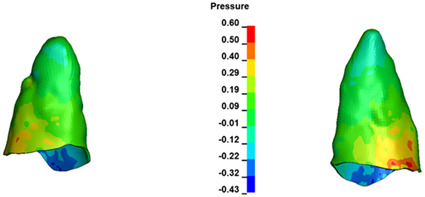
	h = 2 mm	h = 4 mm
	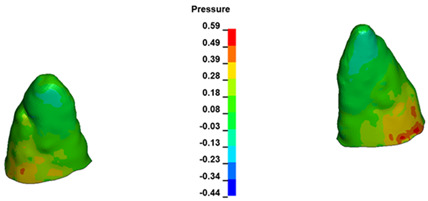	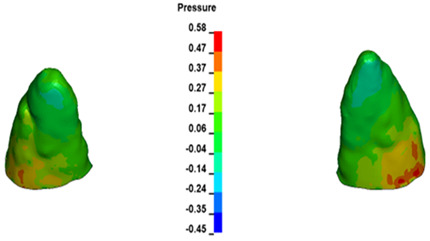
	h = 6 mm	h = 8 mm
	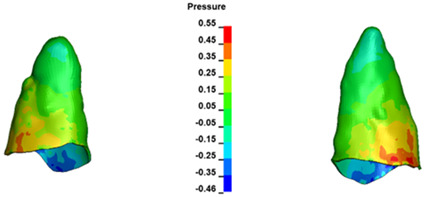	
	h = 10 mm	
150 g	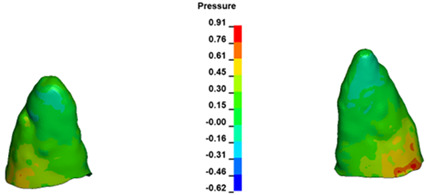	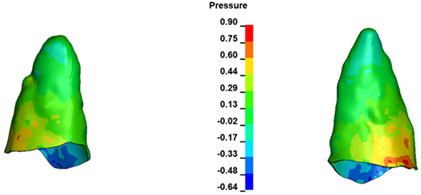
	h = 2 mm	h = 4 mm
	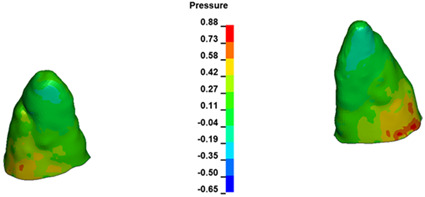	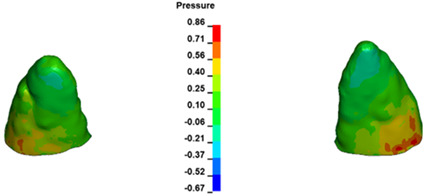
	h = 6 mm	h = 8 mm
	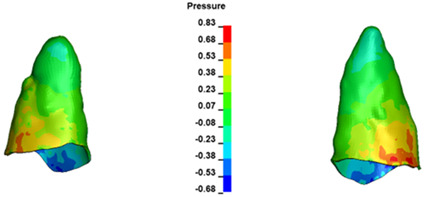	
	h = 10 mm	
200 g	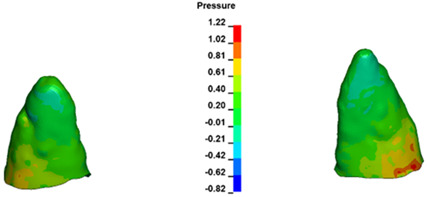	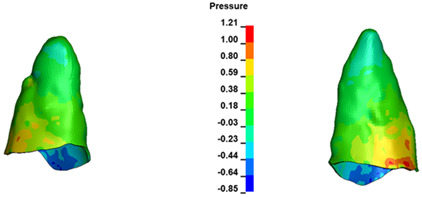
	h = 2 mm	h = 4 mm
	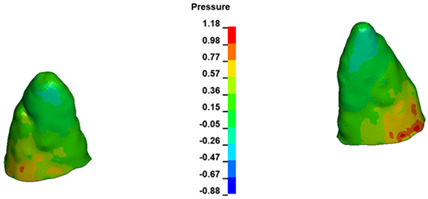	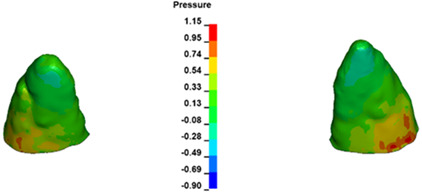
	h = 6 mm	h = 8 mm
	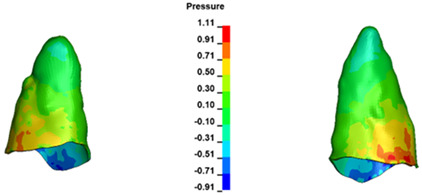	
	h = 10 mm	
300 g	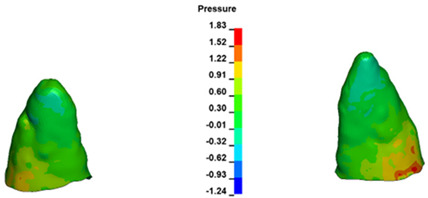	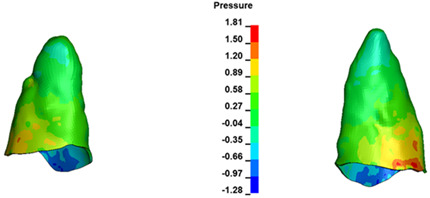
	h = 2 mm	h = 4 mm
	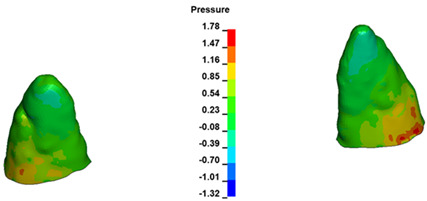	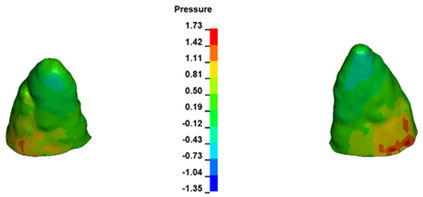
	h = 6 mm	h = 8 mm
	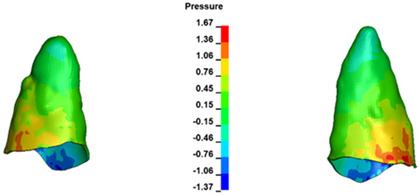	
	h = 10 mm	

**Table 7:** Pressure σ_h_ [kPa] distribution in the PDL—entire dental arch.

50 g	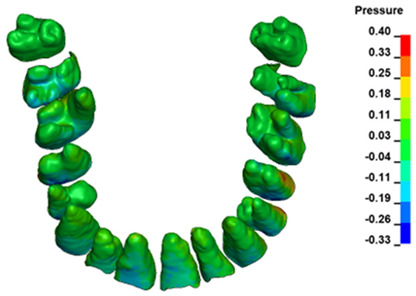	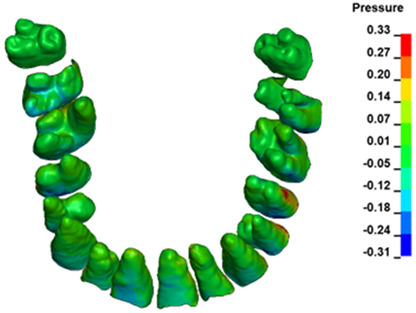
	h = 2 mm	h = 4 mm
	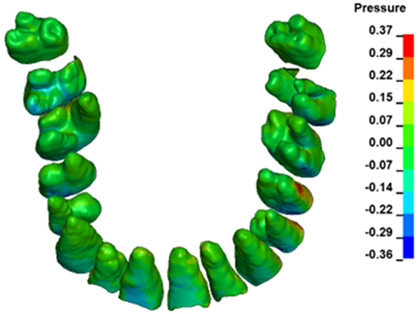	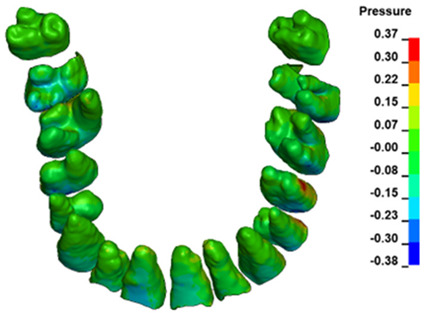
	h = 6 mm	h = 8 mm
	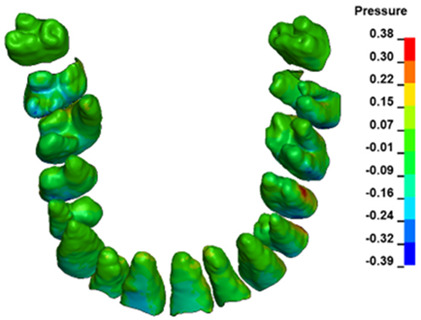	
	h = 10 mm	
100 g	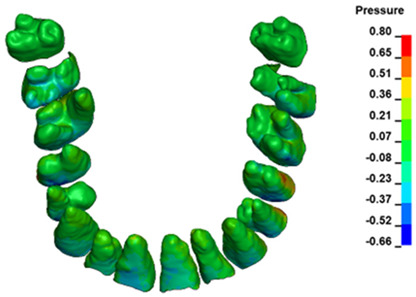	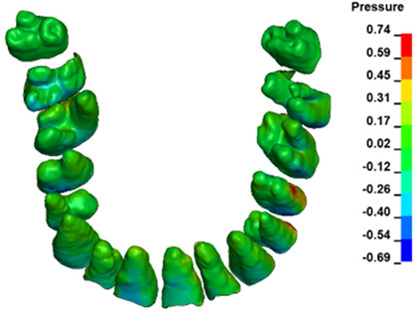
	h = 2 mm	h = 4 mm
	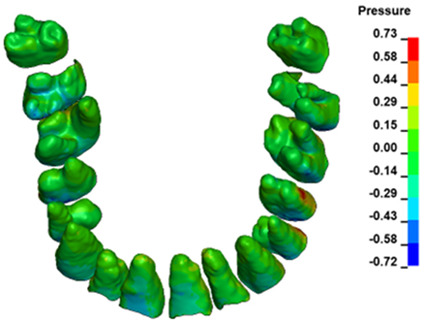	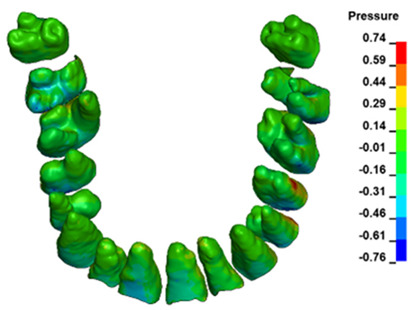
	h = 6 mm	h = 8 mm
	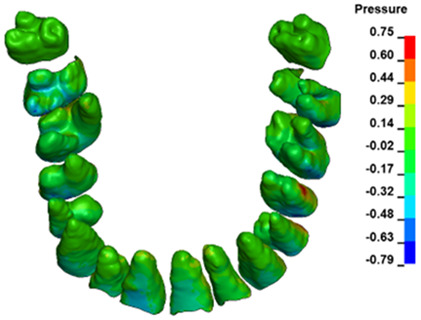	
	h = 10 mm	
150 g	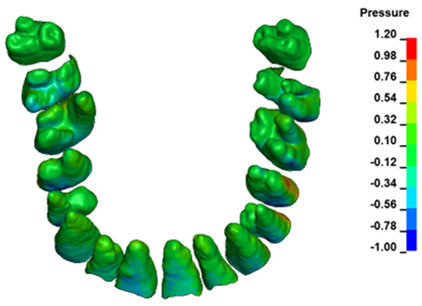	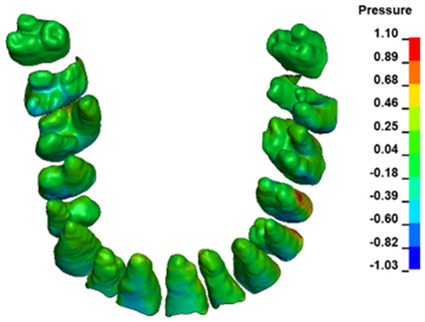
	h = 2 mm	h = 4 mm
	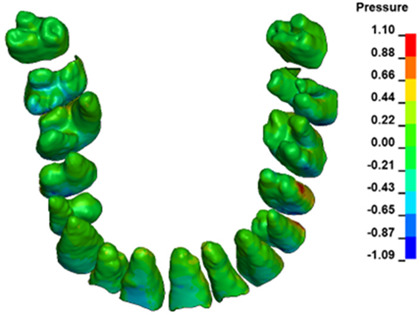	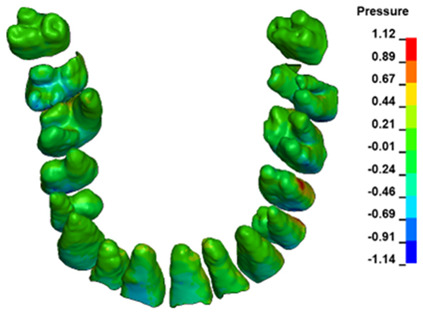
	h = 6 mm	h = 8 mm
	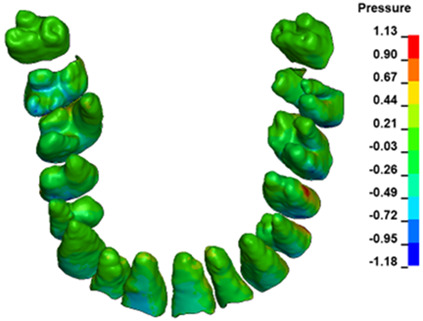	
	h = 10 mm	
200 g	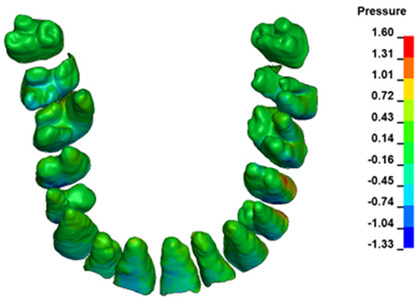	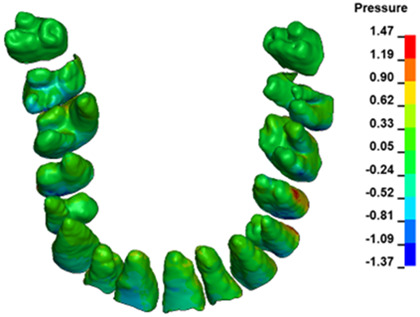
	h = 2 mm	h = 4 mm
	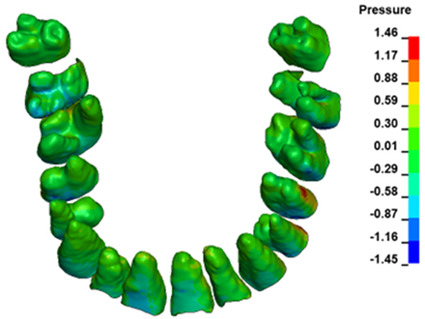	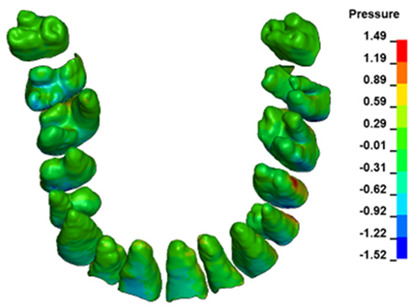
	h = 6 mm	h = 8 mm
	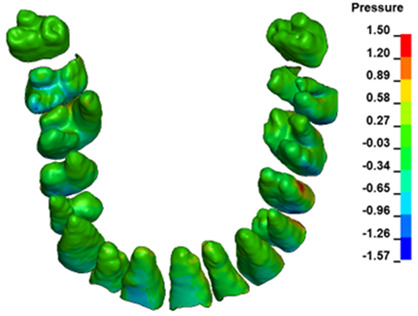	
	h = 10 mm	
300 g	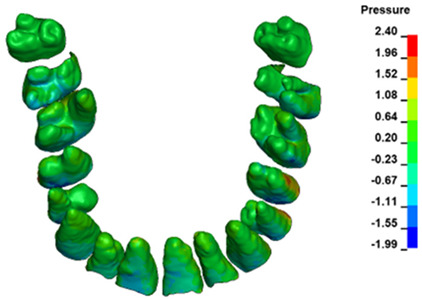	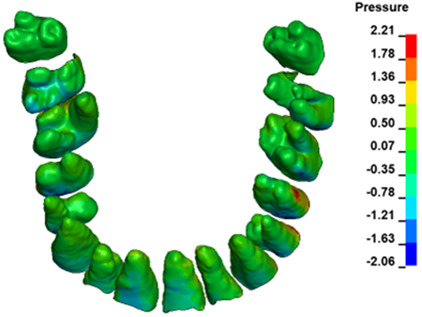
	h = 2 mm	h = 4 mm
	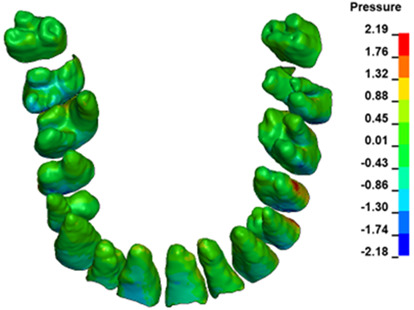	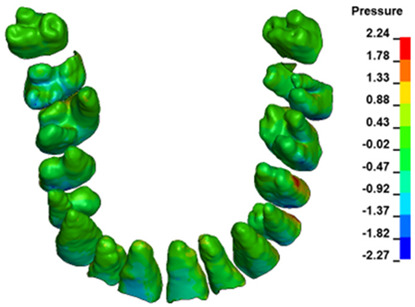
	h = 6 mm	h = 8 mm
	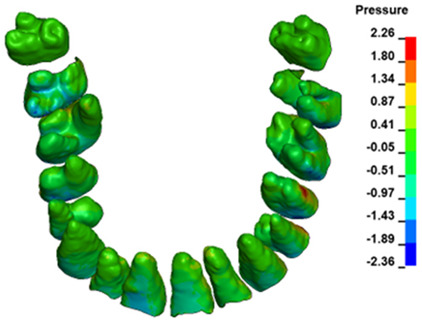	
	h = 10 mm	

**Table 8:** Pressure σ_h_ [kPa] distribution in the PDL—central incisors.

50 g	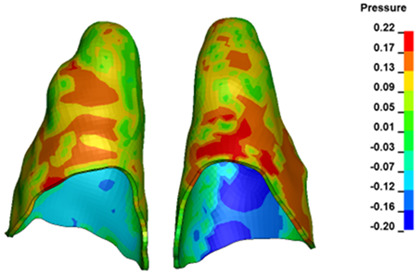	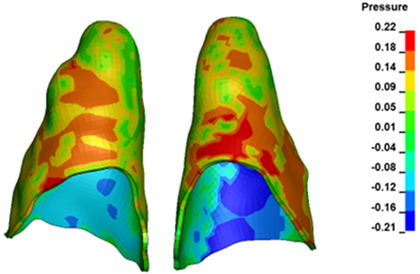
	h = 2 mm	h = 4 mm
	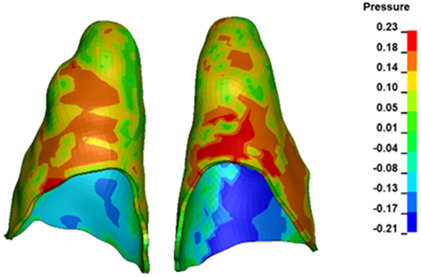	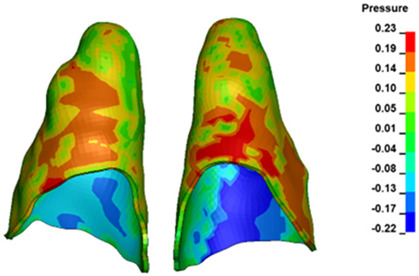
	h = 6 mm	h = 8 mm
	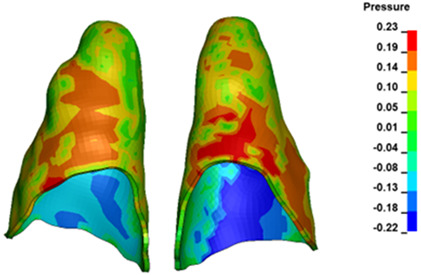	
	h = 10 mm	
100 g	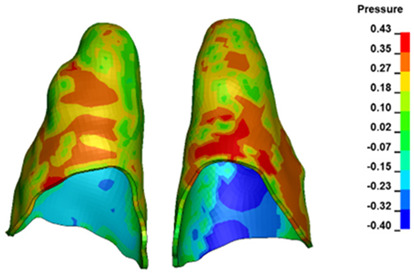	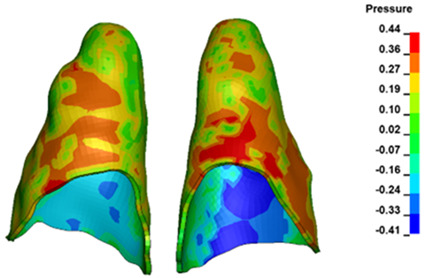
	h = 2 mm	h = 4 mm
	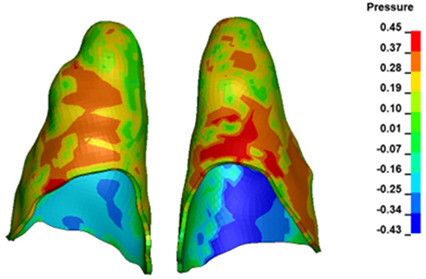	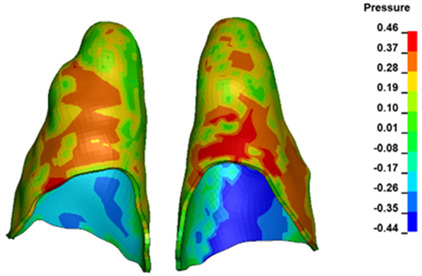
	h = 6 mm	h = 8 mm
	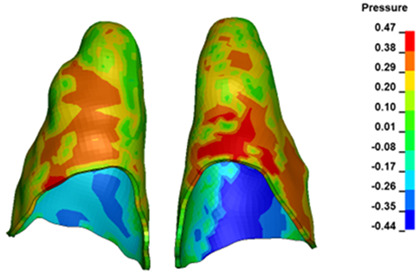	
	h = 10 mm	
150 g	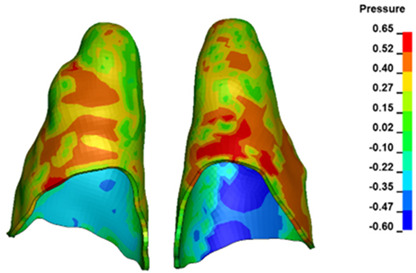	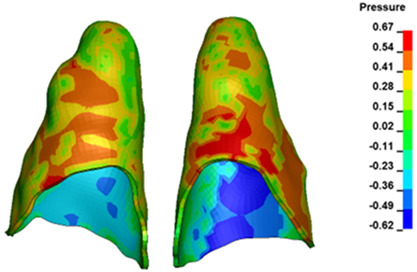
	h = 2 mm	h = 4 mm
	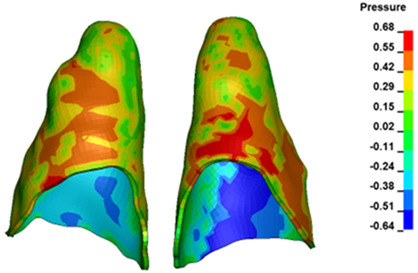	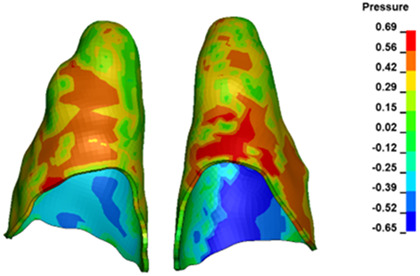
	h = 6 mm	h = 8 mm
	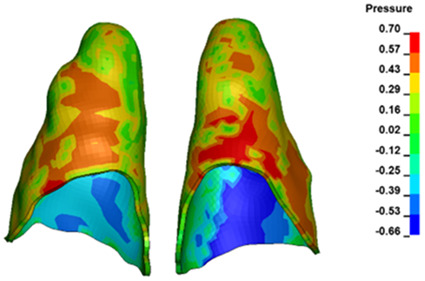	
	h = 10 mm	
200 g	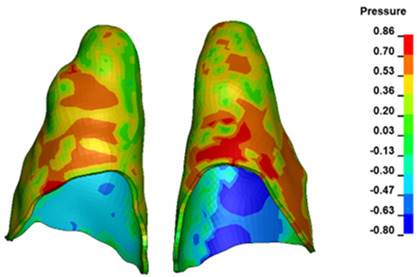	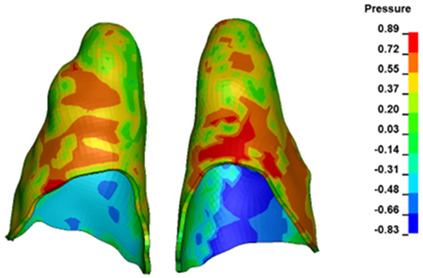
	h = 2 mm	h = 4 mm
	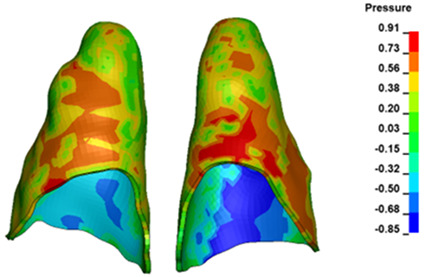	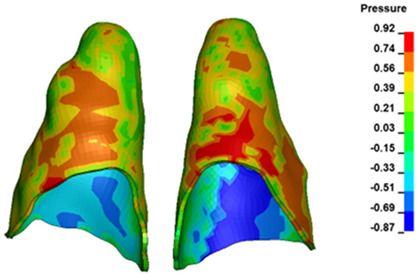
	h = 6 mm	h = 8 mm
	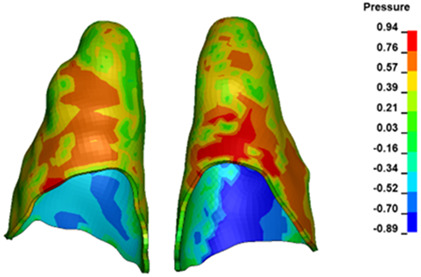	
	h = 10 mm	
300 g	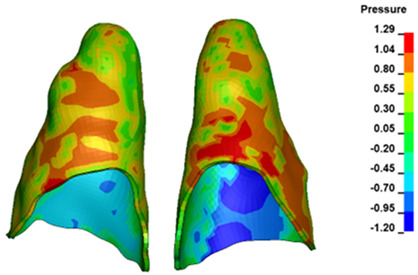	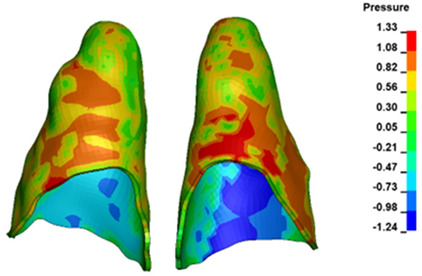
	h = 2 mm	h = 4 mm
	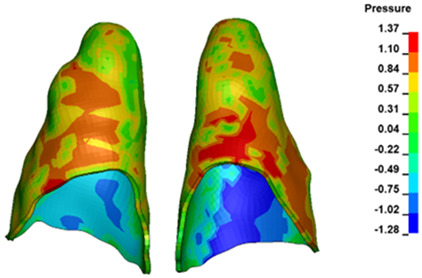	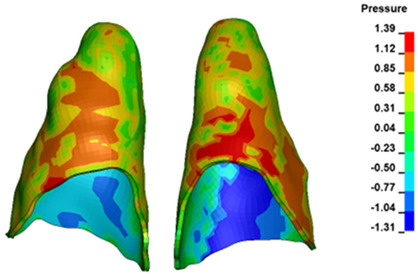
	h = 6 mm	h = 8 mm
	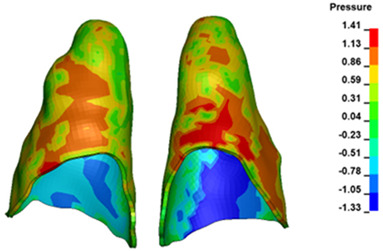	
	h = 10 mm	

**Table 9:** Pressure σ_h_ [kPa] distribution in the PDL—lateral incisors.

50 g	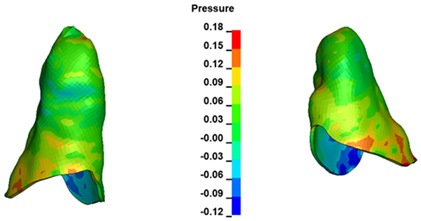	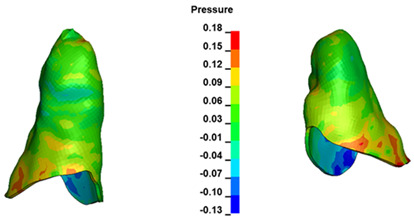
	h = 2 mm	h = 4 mm
	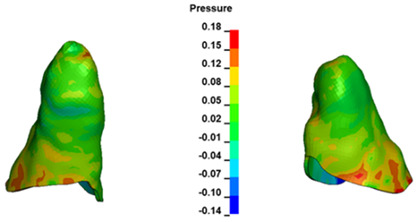	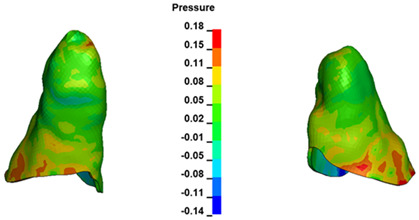
	h = 6 mm	h = 8 mm
	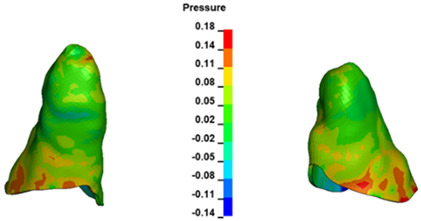	
	h = 10 mm	
100 g	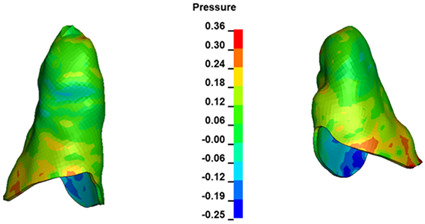	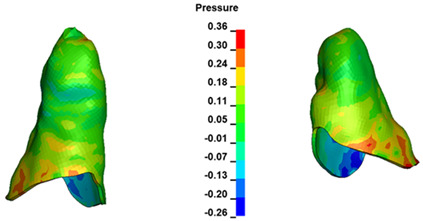
	h = 2 mm	h = 4 mm
	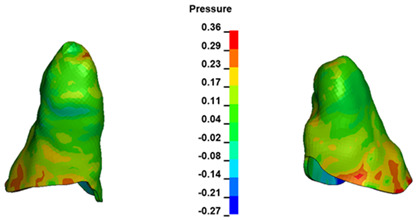	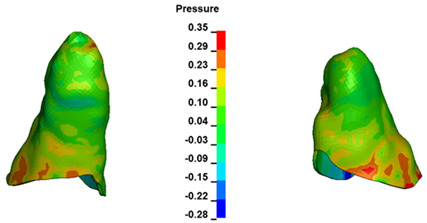
	h = 6 mm	h = 8 mm
	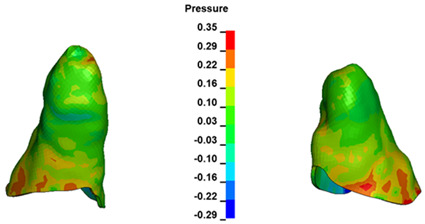	
	h = 10 mm	
150 g	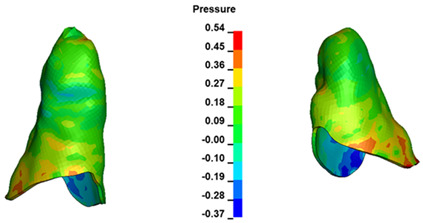	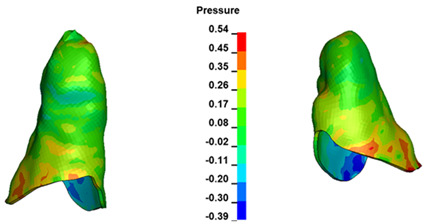
	h = 2 mm	h = 4 mm
	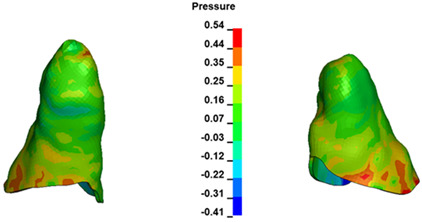	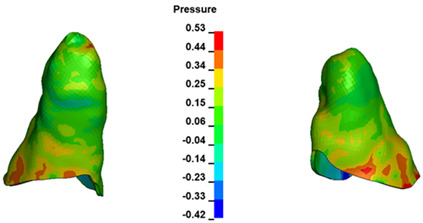
	h = 6 mm	h = 8 mm
	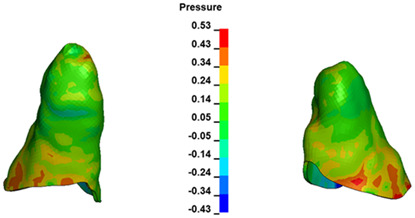	
	h = 10 mm	
200 g	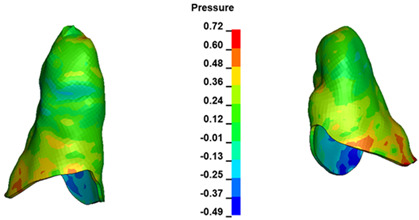	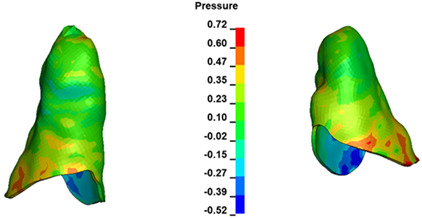
	h = 2 mm	h = 4 mm
	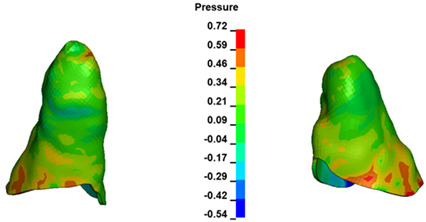	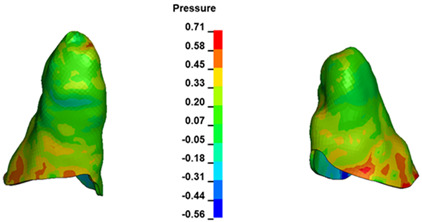
	h = 6 mm	h = 8 mm
	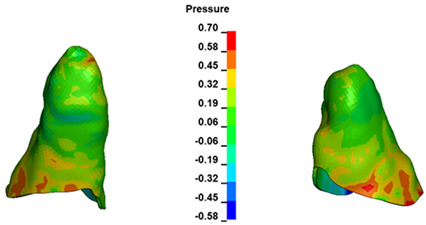	
	h = 10 mm	
300 g	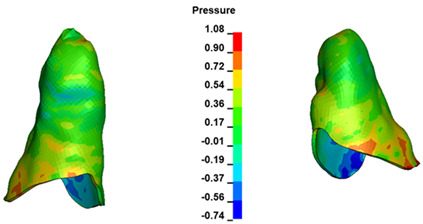	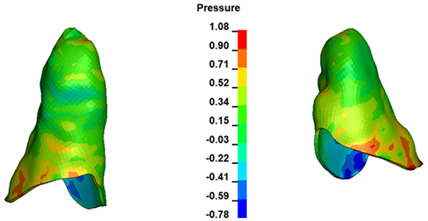
	h = 2 mm	h = 4 mm
	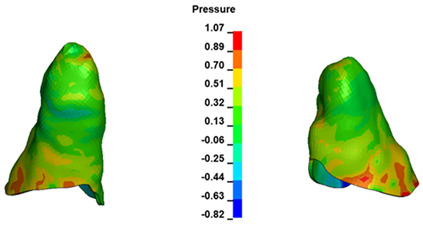	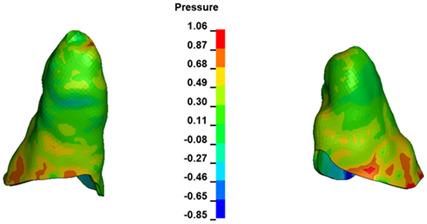
	h = 6 mm	h = 8 mm
	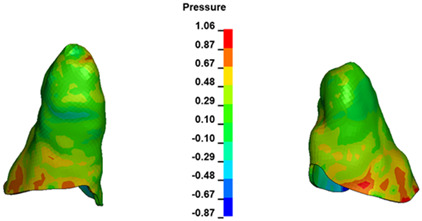	
	h = 10 mm	

**Table 10:** Pressure σ_h_ [kPa] distribution in the PDL—canines.

50 g	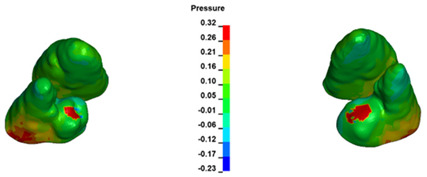	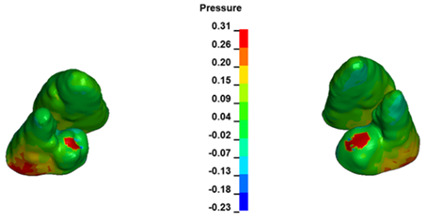
	h = 2 mm	h = 4 mm
	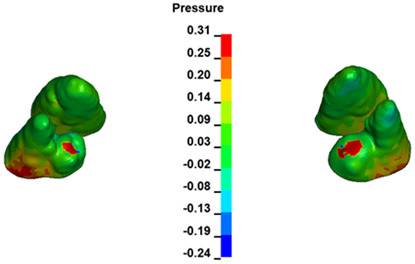	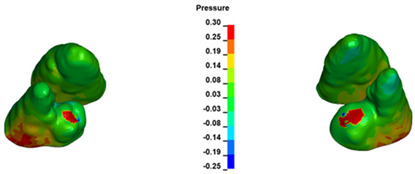
	h = 6 mm	h = 8 mm
	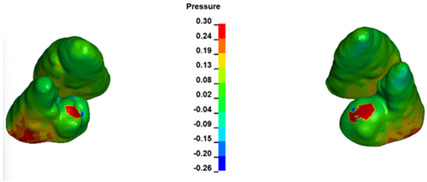	
	h = 10 mm	
100 g	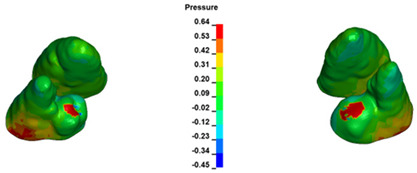	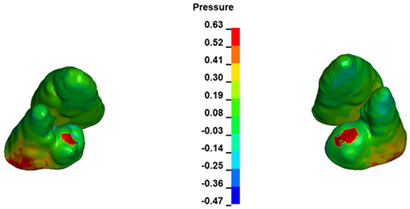
	h = 2 mm	h = 4 mm
	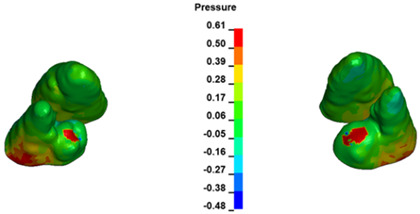	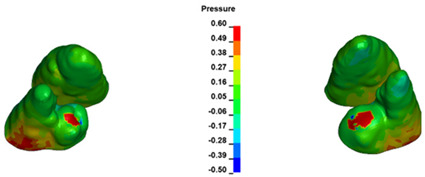
	h = 6 mm	h = 8 mm
	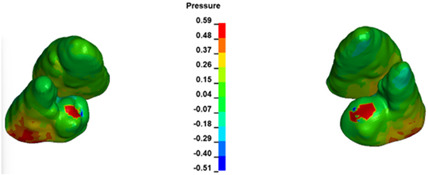	
	h = 10 mm	
150 g	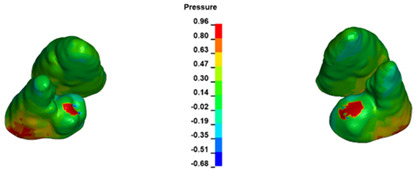	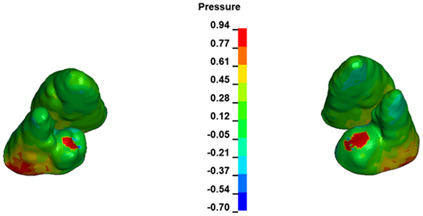
	h = 2 mm	h = 4 mm
	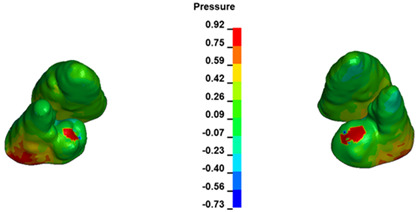	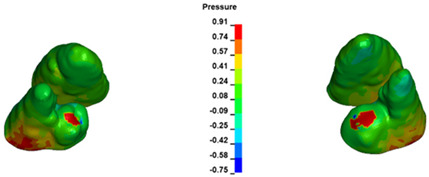
	h = 6 mm	h = 8 mm
	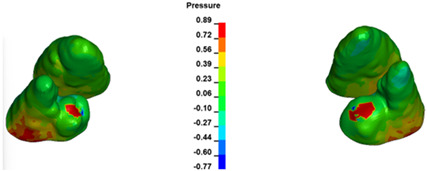	
	h = 10 mm	
200 g	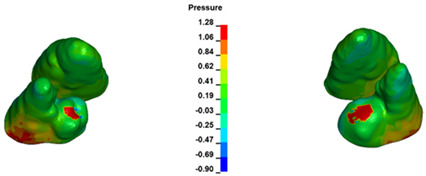	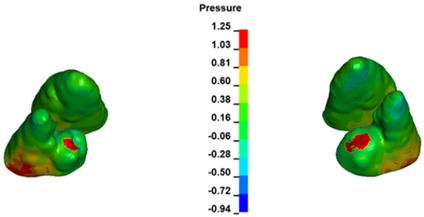
	h = 2 mm	h = 4 mm
	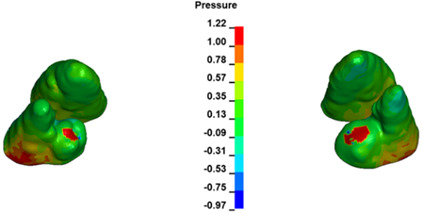	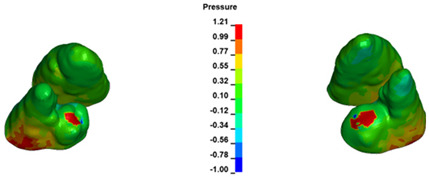
	h = 6 mm	h = 8 mm
	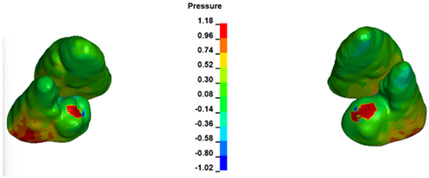	
	h = 10 mm	
300 g	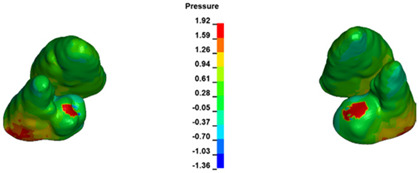	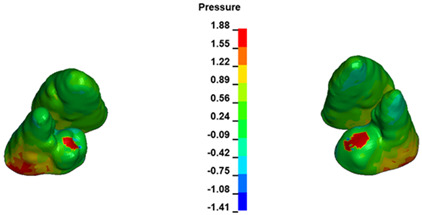
	h = 2 mm	h = 4 mm
	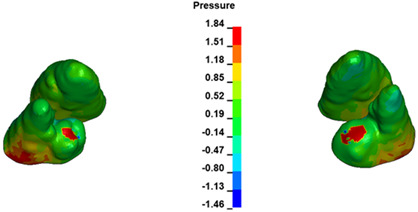	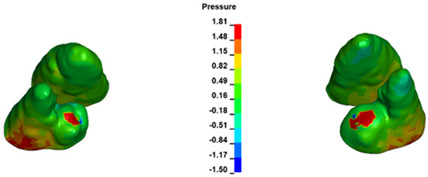
	h = 6 mm	h = 8 mm
	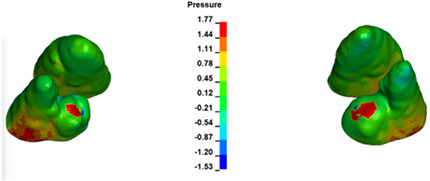	
	h = 10 mm	

## Data Availability

Data are contained within the article.
